# The BAFF/APRIL system in autoimmune nephropathy: from pathogenesis to targeted biologic therapies

**DOI:** 10.3389/fmed.2026.1965501

**Published:** 2026-10-08

**Authors:** Yingjie Jia, Zhenping Yu, Yamin Xue, Guanghui Pan, Xiaoyu Jiang, Farong Zhang

**Affiliations:** 1 College of First Clinical Medicine, Shandong University of Chinese Medicine, Jinan, Shandong, China; 2 Department of Nephrology, First Affiliated Hospital of Shandong University of Chinese Medicine, Jinan, Shandong, China

**Keywords:** April, BAFF, biologics, IgA nephropathy, lupus nephritis, primary membranous nephropathy

## Abstract

BAFF and APRIL, members of the tumor necrosis factor (TNF) superfamily, regulate not only B-cell survival, differentiation, and antibody production but also the differentiation of T-cell subsets through binding to their receptors BAFF-R, TACI, and BCMA. In autoimmune nephropathies, aberrant activation of the BAFF/APRIL signaling pathway drives autoreactive B cells to produce pathogenic autoantibodies, including Gd-IgA1, anti-dsDNA, and anti-PLA2R, which subsequently form immune complexes, deposit in the kidney, and trigger renal tissue injury. Although conventional immunosuppressive therapies can control disease progression to some extent, their adverse effects remain a concern that cannot be overlooked. In recent years, biologic agents targeting this pathway, such as belimumab and telitacicept, have demonstrated favorable efficacy and are gradually being integrated into clinical practice. This review focuses on IgA nephropathy, lupus nephritis, and primary membranous nephropathy, systematically summarizing the pathogenic mechanisms of the BAFF/APRIL axis and the corresponding biologic therapies in these three disease entities, while also discussing current Challenges and Future Perspectives. Of note, in analyzing the available clinical data, we observed that APRIL appears to be predominate in IgA nephropathy, whereas BAFF targeted strategies are more frequently employed in lupus nephritis and primary membranous nephropathy. These differences, however, are based on cross-trial comparisons and should be regarded as a preliminary working hypothesis requiring validation by direct head-to-head trials.

## 1 Introduction

Autoimmune nephropathy is a group of diseases in which dysfunction of the immune system leads to the production of autoantibodies that cause damage to kidney tissue. Clinically, it commonly manifests as proteinuria, hematuria, and progressive decline in renal function, with some patients progressing to end-stage renal disease, posing a serious threat to life and health. Current Western medical treatment primarily relies on glucocorticoids and immunosuppressants. However, long-term use is associated with adverse effects such as infections and metabolic disorders. Additionally, some patients experience limited therapeutic efficacy, high recurrence rates, and drug resistance (1, 2). There is an urgent clinical need for safer and more effective intervention strategies.

B-cell activating factor (BAFF) and a proliferation-inducing ligand (APRIL) are key members of the tumor necrosis factor superfamily and serve as critical regulators of B-cell survival, differentiation, and antibody production. By binding to BAFF receptor(BAFF-R), transmembrane activator and calciummodulator and cyclophilin ligand interactor(TACI), and B-cell maturation antigen(BCMA) receptors, their overexpression can disrupt immune homeostasis and drive aberrant activation of autoreactive B cells, thereby playing a pivotal role in the pathogenesis of autoimmune nephropathy (3, 4). Aberrantly elevated levels of BAFF/APRIL can induce autoreactive B cells to escape tolerance and differentiate into plasma cells, which continuously secrete pathogenic antibodies such as anti-dsDNA and anti-PLA2R. These antibodies form immune complexes that deposit in the glomeruli, activating complement and inflammatory pathways and causing renal damage (5, 6). Concurrently, BAFF regulates the differentiation of T cell subsets such as follicular helper T cells (Tfh) and T helper cell(Th)17 cells and suppresses eegulatory T cell(Treg) function, further amplifying the autoimmune inflammatory response (7, 8).

In recent years, targeting BAFF/APRIL has emerged as a leading therapeutic approach for autoimmune nephropathies. For instance, the anti-BAFF monoclonal antibody (belimumab) has been approved for the treatment of lupus nephritis (9), and the TACI-Fc fusion protein (telitacicept), which simultaneously blocks BAFF and APRIL, has demonstrated renal remission benefits in both lupus nephritis and IgA nephropathy (10, 11). These strategies block the activation of pathogenic B cells and the production of autoantibodies at the upstream level, offering a new mechanism-based therapeutic paradigm for autoimmune nephropathy. The treatment of autoimmune nephropathy has shifted from traditional symptomatic anti-inflammatory therapy to precision targeted therapy.

Given the central role of the BAFF/APRIL pathway in autoimmune kidney diseases and the substantial therapeutic potential of its targeted intervention, this review summarizes the pathogenic mechanisms of BAFF and APRIL, outlines the clinical progress of biologic agents targeting this pathway, and discusses current challenges as well as future perspectives. Our aim is to facilitate further exploration in this field and to optimize clinical practice.

## 2 BAFF/APRIL and immune cell regulation

### 2.1 Biological effects of BAFF/APRIL on B cells

BAFF and APRIL are key members of the tumor necrosis factor (TNF) superfamily. Both are synthesized as type II transmembrane proteins and, following cleavage by proteases (such as furin), form soluble homotrimers that exert their biological effects. They serve as critical factors for B lymphocyte survival, maturation, and differentiation into plasma cells (12, 13). Together, they regulate B-cell homeostasis through interactions with B-cell surface receptors (BAFF-R, TACI, and BCMA). As a central factor in B-cell activation and development, BAFF is essential for the survival of immature B cells and the differentiation of transitional B cells into mature B cells, whereas APRIL focuses more on promoting the proliferation of mature B cells and the survival and antibody secretion of plasma cells, playing a particularly crucial role in maintaining long-term humoral immune memory (14, 15). Notably, BAFF can bind to three receptors—BAFF-R, TACI, and BCMA—while APRIL binds only to TACI and BCMA, but not to BAFF-R (16). Furthermore, APRIL also interacts with proteoglycans (heparan sulfate proteoglycans, HSPGs) as a coreceptor, influencing its local tissue bioavailability (17, 18). Studies have shown that the expression of these three receptors differs markedly depending on the stage of B-cell development. BAFF-R is primarily expressed in immature B cells, mediating their survival and maturation. As B cells differentiate into plasma cells and memory B cells, BAFF-R expression is significantly downregulated, whereas TACI and BCMA are gradually upregulated. Among these, BAFF-R is an essential receptor in B-cell development, primarily mediating the survival and maturation of immature B cells, transitional B cells, and early mature B cells. TACI is a key receptor regulating B-cell class switching. For example, in IgA nephropathy, TACI binds to APRIL/BAFF to drive B-cells toward IgA class switching, thereby producing pathogenic galactose-deficient IgA1 (Gd-IgA1). BCMA is preferentially expressed by long-lived plasma cells (PCs) and naïve or memory B cells, maintaining the functional stability of plasma cells. Together, these three receptors ensure the orderly initiation, efficient execution, and long-term maintenance of the humoral immune response (4, 19–21) (Figure 1).

**Figure 1:**
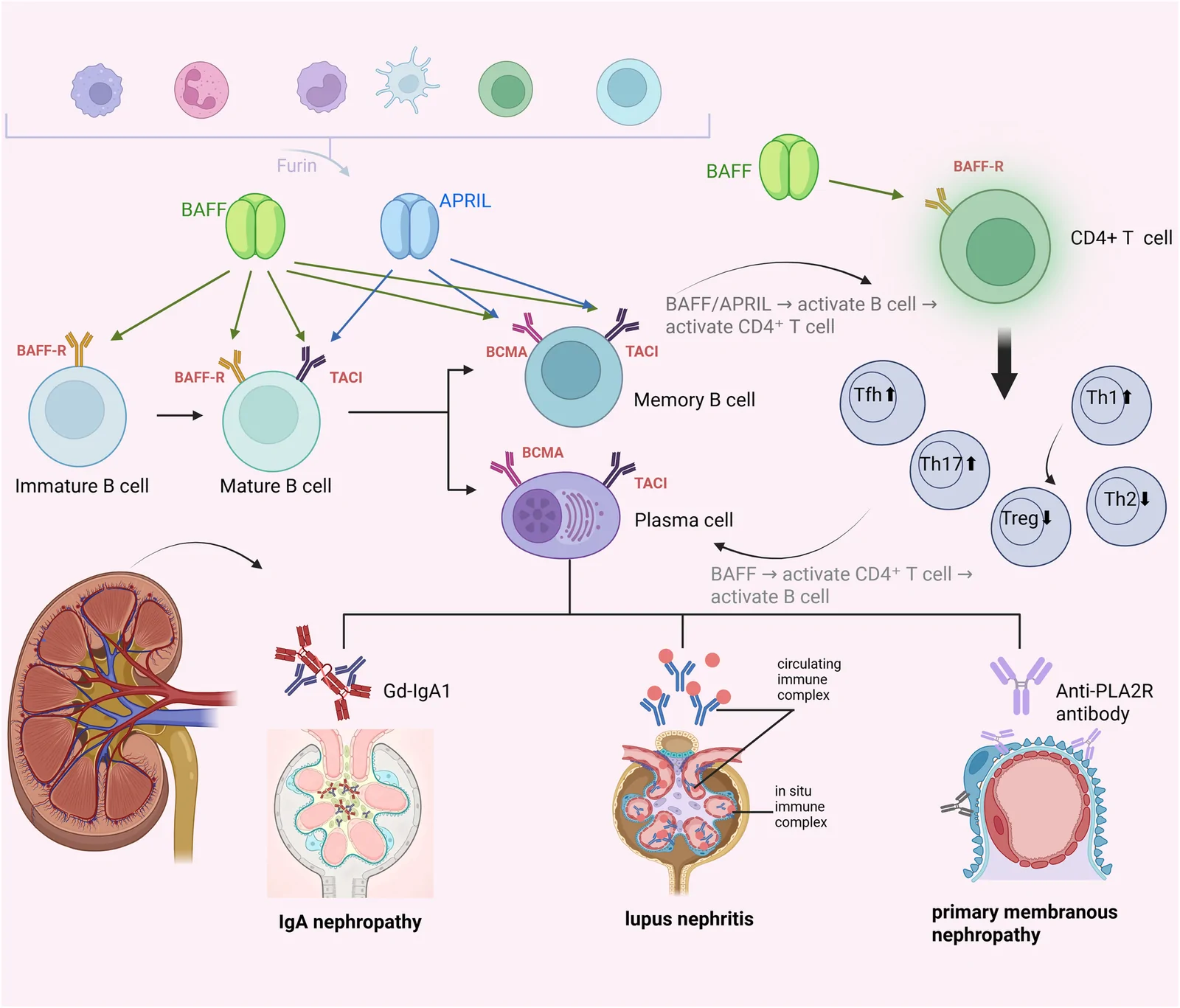
Mechanism of BAFF/APRIL-induced autoimmune nephropathy: BAFF and APRIL form soluble trimers following cleavage by furin; through differential binding to BAFF-R, TACI, and BCMA receptors, they regulate B-cell survival, maturation, and differentiation. Concurrently, BAFF can directly activate CD4⁺ T cells, promote Tfh differentiation, and upregulate the Th1/Th2 and Th17/Treg ratios, thereby further enhancing the survival, differentiation, and antibody production of autoreactive B cells. It is involved in the pathogenesis of immune complex-associated nephropathies such as IgA nephropathy, lupus nephritis, and primary membranous nephropathy. Created with BioRender.

Importantly, plasma cells are not a uniform population. Following antigen exposure, activated B cells initially differentiate into short-lived plasmablasts, which rapidly secrete antibodies but persist for only a few days. In contrast, long-lived plasma cells (LLPCs) can continue secreting antibodies for years or even decades and represent the major source of sustained pathogenic autoantibody production (22, 23). Short-lived plasmablasts rely on BAFF, whereas LLPCs reside primarily in the bone marrow (BM) and depend mainly on APRIL/BCMA signaling, rather than BAFF (15, 24–26). In addition, LLPC survival depends on tissue heparan sulfate proteoglycans (HSPGs), which concentrate APRIL locally to maintain a high concentration that supports plasma cell survival (27, 28). This distinction has important clinical implications. Selective BAFF blockade primarily depletes immature and transitional B cells and reduces the generation of new plasmablasts, but it struggles to eliminate established, class-switched LLPCs that have acquired a BCMA-dependent survival program. In contrast, anti-APRIL antibodies and TACI-Fc fusion proteins interrupt APRIL–BCMA/TACI signaling, thereby more directly targeting the LLPC pool and reducing pre-existing pathogenic autoantibody titers. This distinction is crucial for understanding why selective anti-BAFF monoclonal antibodies (e.g., belimumab) struggle to deplete established autoantibody titers in pMN or IgAN compared to TACI-Fc fusion proteins or anti-APRIL agents (29, 30). This also suggests that therapeutic strategies aimed at achieving durable remission should consider not only suppressing the generation of new autoreactive B cells but also eliminating or silencing the LLPC reservoir.

### 2.2 The immunoregulatory effects of BAFF/APRIL on T cells

In addition to their classic role in B-cell regulation, BAFF and APRIL are also involved in T-cell immune regulation. BAFF regulates the differentiation of CD4+ T cells—particularly Tfh and Th17 cells—by binding to the BAFF receptor (BR3) on the T-cell surface, thereby further supporting B-cell function. In contrast, APRIL plays no significant role in this process (8, 31, 32). Furthermore, in transgenic mouse models, it has been found that BAFF also participates in regulating the balance between Th1 and Th2 subsets and inhibits the differentiation of Treg (7, 33). Studies have confirmed that Th1/Th2 immune imbalance can indirectly affect B-cell activation and function, thereby contributing to the pathological process of autoimmune diseases. In particular, excessive Th1 activation can amplify pro-inflammatory cytokine storms, suppress anti-inflammatory pathways such as Th2, Treg, and Breg (IL-10+ B cells), and disrupt immune tolerance (34, 35). Concurrently, dysregulation of regulatory T cells (Tregs) is also a key mechanism in the onset and progression of autoimmune diseases. In systemic lupus erythematosus (SLE), reduced Treg numbers and impaired function hinder the effective suppression of autoreactive T cell activation and abnormal B cell proliferation, ultimately leading to the massive production of pathogenic autoantibodies and contributing to disease progression (36). Therefore, an imbalance in CD4^+^ T cell subsets is a key upstream mechanism driving abnormal B cell activation. T cells can enhance the survival, differentiation, and antibody production of autoreactive B cells, and promote inflammatory responses and tissue damage through the secretion of cytokines (37). Taken together, blockade of the BAFF/APRIL system affects multiple immune cell populations, and targeting the BAFF signaling pathway may interact with B cells and multiple T cell subsets (Figure 1).

Furthermore, two specialized T-cell subsets, Tfh and T follicular regulatory (Tfr) cells, play critical roles in the initiation, maintenance, and regulation of germinal center (GC) responses (38, 39). BAFF-R signaling in T cells modulates the balance between Tfh and Tfr cells within germinal centers and influences B-cell class-switch recombination (CSR). Specifically, when BAFF binds to BAFF-R on T cells, it promotes Tfh cell development, GC formation, and Tfh cell accumulation (8, 40). Tfh cells drive CSR in B cells by providing helper signals such as CD40L, IL-4, and IL-21. Conversely, Tfr cells suppress Tfh responses and GC reactions, including GC B-cell differentiation, antibody affinity maturation, and plasma cell differentiation, thereby indirectly limiting CSR (41, 42). Disruption of the Tfh/Tfr balance has been associated with the development of multiple autoimmune diseases (43, 44). Although direct evidence that BAFF-R signaling in T cells regulates Tfr cells is currently lacking, BAFF-R–dependent expansion of Tfh cells may alter the local Tfh/Tfr ratio within germinal centers, thereby shifting the relative strength of Tfh-derived helper signals and Tfr-derived suppressive signals, and ultimately influencing CSR. This suggests that targeting BAFF signaling may represent a potential strategy for modulating the Tfh/Tfr balance and treating autoimmune diseases. However, this hypothesis remains to be validated experimentally.

Besides affecting B cells and T cells, BAFF/APRIL can also promote the differentiation of immune cells such as macrophages and dendritic cells, enhance the secretion of inflammatory cytokines, and disrupt immune balance (45, 46). BAFF and APRIL are primarily produced by myeloid cells, including dendritic cells (cDCs), monocytes, neutrophils, and macrophages, while their expression levels in activated B cells and T cells are relatively low (47, 48). Studies have shown that the expression of BAFF and APRIL can be significantly upregulated by stimuli such as interleukin (IL)-10, IL-4, IFN-*γ*, Toll-like receptor (TLR) agonists (TLR4, TLR9), and transforming growth factor-*β* (TGF-*β*) (49).

In summary, the BAFF/APRIL system regulates a variety of biological processes and plays a key regulatory role in B-cell development, differentiation, and antibody production, as well as in the differentiation of T-cell subsets. A thorough understanding of the origins and expression regulation of BAFF/APRIL, as well as the specific roles they play in B-cell and T-cell biology, is crucial for the development of targeted therapies for autoimmune diseases.

## 3 The role of BAFF/APRIL in autoimmune nephropathy

Autoimmune nephropathy is a group of diseases caused by dysfunction of the immune system, in which the body mistakenly attacks its own kidney tissue, leading to kidney damage. It is typically characterized by excessive activation of B cells and T cells (6, 50). The BAFF/APRIL system primarily targets B cells to drive autoimmune responses by binding to three receptors-BAFF-R, TACI, and BCMA-to transmit anti-apoptotic and pro-differentiation signals. In renal autoimmunity, excessive BAFF and APRIL allow auto-reactive B cells to evade bone marrow tolerance checkpoints, promote their survival, and, with T-cell assistance in the germinal center, differentiate into plasma cells and long-lived plasma cells that continuously secrete pathogenic autoantibodies (such as anti-dsDNA, anti-PLA2R, and anti-GBM antibodies). These antibodies form *in situ* immune complexes in the glomeruli, activating complement and causing renal damage (20).

In this process, T cells are by no means mere bystanders. As a costimulatory factor, BAFF acts directly on activated T cells, promoting their proliferation, differentiation, and secretion of pro-inflammatory factors, such as differentiation into follicular helper T cells (Tfh). Tfh cells bind to B-cell CD40 via CD40L, providing critical co-stimulatory signals for the germinal center response and ensuring the affinity maturation and class switching of autoantibodies (51, 52). Concurrently, high concentrations of BAFF can suppress the function of regulatory T cells (Tregs), thereby weakening the negative regulation of autoimmunity (36). It is precisely this abnormal synergy between B cells and T cells that collectively establishes the immunological microenvironment conducive to the production of pathogenic autoantibodies, making the kidneys a target organ for immune attack (Figure 1).

Recent advances in understanding the pathogenic mechanisms of BAFF/APRIL have spurred the development of targeted therapeutic approaches. Multiple novel biologics have entered clinical practice, serving as valuable adjuncts to conventional treatment. Below, we provide a summary and analysis of clinical trial findings regarding BAFF/APRIL-targeted biologic agents for the treatment of autoimmune nephropathy.

## 4 Biologic therapies targeting BAFF/APRIL in autoimmune nephropathy

Multiple clinical trials have demonstrated that blockade of the BAFF/APRIL signaling pathway confers substantial therapeutic benefits in the treatment of autoimmune kidney diseases. The following discussion will focus on IgA nephropathy, lupus nephritis, and primary membranous nephropathy, with a detailed elaboration on the pathogenic mechanisms mediated by the BAFF/APRIL axis, and an overview of emerging biologic agents that target this pathway. The biologic agent–related findings were derived from clinical trials registered on ClinicalTrials.gov (Table 1 and Figure 2). Detailed trial parameters (inclusion/exclusion criteria, baseline characteristics, intervention regimen, primary and key secondary endpoints) from completed or interim-reported studies of BAFF/APRIL-targeted agents in IgAN, LN, and pMN are provided in Supplementary Table 1.

**Table 1:** Targeting BAFF/APRIL biologics in IgAN, LN, pMN.

BAFF/APRIL-targeted	Biologics	Disease	Trial registration number	Number of participants	Status	Phase	Reference
BAFF	Blisibimod	IgAN	NCT02062684	57	Completed	Phase 2/3	(61)
	Belimumab	LN	NCT01639339	448	Completed	Phase 3	(87)
			NCT02260934	43	Completed	Phase 2	(88)
		pMN	NCT01610492	14	Completed	Phase 2	(29)
			NCT03949855	58	Recruiting	Phase 2	(102)
APRIL	Sibeprenlimab	IgAN	NCT03719443	51	Completed	Phase 1	(64)
			NCT04287985	155	Completed	Phase 2	(65)
			NCT05248646	530	Active, not recruiting	Phase 3	(60)
	Zigakibart		NCT03945318	103	Completed	Phase 1/2	(66)
			NCT05852938	383	Active, not recruiting	Phase 3	(67)
BAFF/APRIL	Telitacicept	IgAN	NCT05799287	318	Active, not recruiting	Phase 3	(69)
			NCT04291781	44	Completed	Phase 2	(70)
		LN	NCT05680480	120	Recruiting	Phase 2	(91)
			NCT07031674	56	Completed	/	(92)
		pMN	NCT06614985	172	Not yet recruiting	Phase 2	(103)
	Atacicept	IgAN	NCT04716231	376	Active, not recruiting	Phase 3	(76)
			NCT02808429	16	Terminated(sponsor decision-unexpectedly slow enrollment)	Phase 2	(75)
		LN	NCT00573157	6	Terminated(due to unanticipated safety issues)	Phase 2/3	(93)
		pMN	NCT06983028	250	Recruiting	Phase 2	(104)
	Povetacicept	IgAN/pMN	NCT05732402	72	Active, not recruiting	Phase 1/2	(30)
		IgAN	NCT06564142	605	Active, not recruiting	Phase 3	(77)

**Figure 2:**
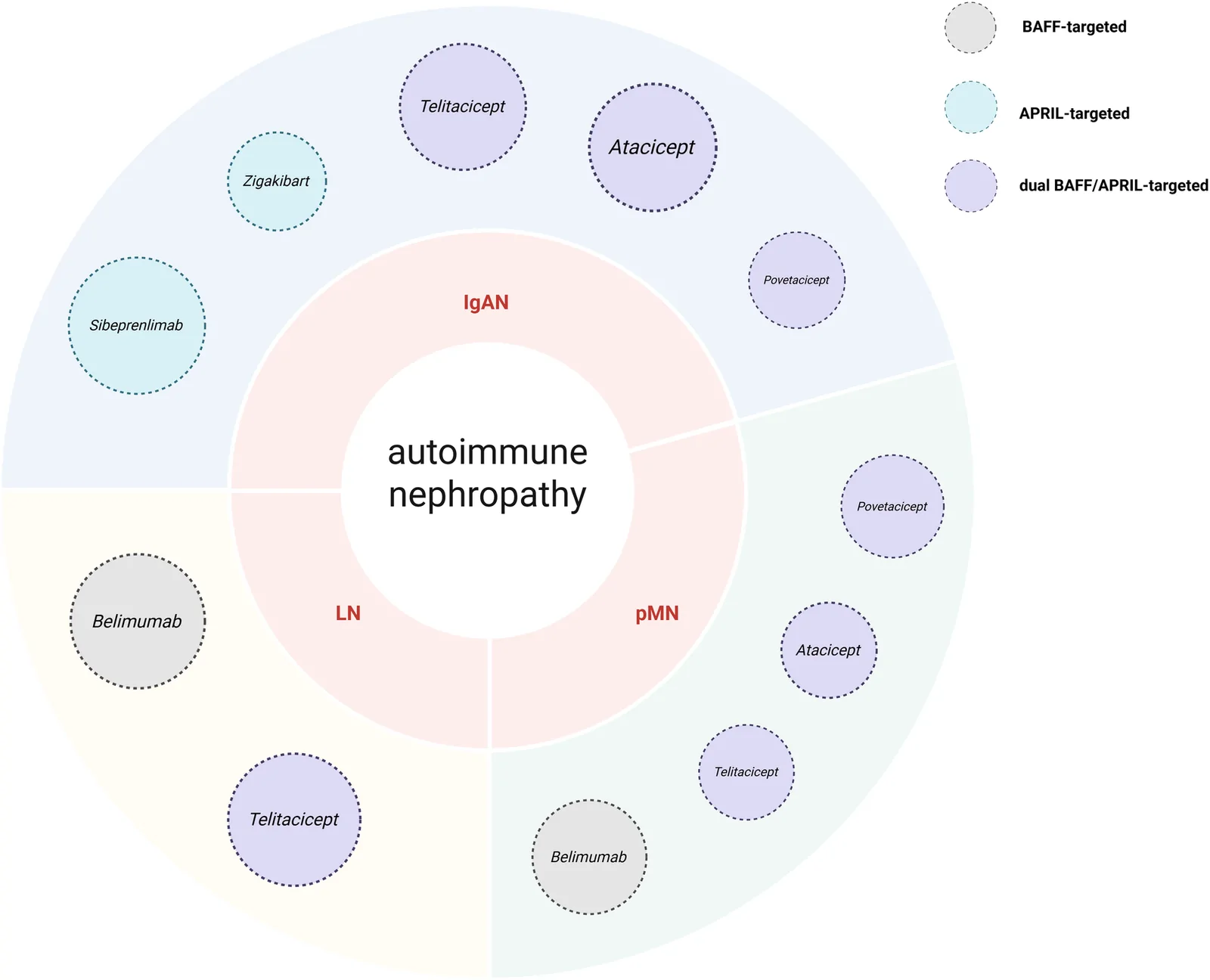
BAFF/APRIL-targeting biologics currently under clinical trials for IgAN, LN, and pMN. A larger circle indicates more advanced clinical development of the biologic in that disease.

### 4.1 Iga nephropathy

#### 4.1.1 BAFF/APRIL in the pathogenesis of igA nephropathy

IgA nephropathy (IgAN) is the most common form of primary glomerulonephritis worldwide. Its pathogenesis is complex, with the immune process primarily characterized by abnormal B-cell activation, accompanied by T-cell immune dysregulation (53). Clinically, treatment typically involves corticosteroids and immunosuppressants. However, long-term use is associated with numerous adverse effects, and many patients still progress to end-stage renal disease (54, 55). Existing research confirms that the abnormal production of Gd-IgA1 is the central initiating step in the pathogenesis of IgAN and the key origin of the “quadruple hit” hypothesis (56). BAFF and APRIL promote B-cell activation and abnormal differentiation by binding to TACI, BCMA, and BAFF-R receptors, driving excessive production of Gd-IgA1. This, in turn, facilitates immune complex deposition and renal tissue damage, accelerating the progression of IgAN. Among these, APRIL promotes IgA class switching in plasma cells, strongly driving the production of Gd-IgA1 (57). Furthermore, BAFF can activate the PI3K-Akt signaling pathway via BAFF-R, thereby contributing to the regulation of T-cell activation and proliferation and further amplifying autoimmune inflammatory responses (58). Currently, biologics targeting BAFF/APRIL (such as sibeprenlimab and atacicept) have entered the clinical trial phase and demonstrated promising efficacy, significantly alleviating clinical symptoms and slowing disease progression in patients with IgA nephropathy, thereby offering a novel therapeutic strategy for IgAN (59, 60). Consequently, BAFF/APRIL has recently emerged as a new therapeutic target in the management of IgA nephropathy.

#### 4.1.2 BAFF/APRIL-targeted biologics

Blisibimod Blisibimod is a selective BAFF inhibitor that acts by modulating B-cell activity through BAFF blockade, thereby potentially reducing the production of Gd-IgA. In a randomized, double-blind, placebo-controlled Phase 2/3 study of blisibimod (BRIGHT-SC), the prespecified primary statistical endpoint (complete or partial remission of proteinuria) was not met at Week 48. Anthera Pharmaceuticals permitted continued dosing for up to two years. Extended treatment suggested trends toward proteinuria stabilization and eGFR preservation (+6.2 vs. −4.8 mL/min/1.73 m^2^/year for placebo), though the trial results have not yet been formally published in a peer-reviewed journal (61). At present, monoclonal antibodies targeting BAFF alone remain at a very early exploratory stage. Given the critical pathogenic role of APRIL in IgA nephropathy, the research focus has shifted from sole BAFF blockade toward APRIL antibody monotherapy and dual inhibition strategies targeting both BAFF and APRIL.

Sibeprenlimab Sibeprenlimab (Voyxact) is a humanized IgG2 monoclonal antibody targeting APRIL. In November 2025, it received accelerated approval from the U.S. Food and Drug Administration (FDA) for reducing proteinuria in adult patients with primary IgA nephropathy at risk of disease progression, making it the first APRIL-targeted agent to obtain global regulatory approval (62, 63). In a randomized, double-blind, single-ascending-dose phase 1 clinical trial, sibeprenlimab (VIS649) demonstrated a favorable tolerability profile and produced dose-dependent, reversible reductions in serum APRIL protein, immunoglobulin levels, and Gd-IgA1 (64). In a subsequent phase 2 clinical trial (NCT04287985), 12-month sibeprenlimab (4 or 8 mg/kg monthly) reduced 24-h UPCR(Urine Protein-to-Creatinine Ratio) by 58.8% and 62.0%, respectively, versus 20.0% with placebo. eGFR changed by +0.2 and −1.5 mL/min/1.73 m^2^, respectively, versus −7.4 with placebo. Clinical remission (UPCR <0.3 g/g) occurred in 12.2% and 26.3% of patients in the 4-mg and 8-mg groups, respectively, versus 2.6% with placebo (65). Consistent with the phase 2 trial results, an interim analysis of a phase 3 study confirmed that sibeprenlimab reduced 24-hour UPCR by 50.2% at 9 months vs. a 2.1% increase with placebo (placebo-adjusted −51.2%; *P* < 0.001). At 12 months, UPCR fell 56.6% vs. 5.1% (difference −54.3%). APRIL and Gd-IgA1 fell by 95.8% and 67.1%, respectively. eGFR slope data remain blinded. Safety was similar between groups (60). However, as the current findings are derived from an interim analysis of the clinical trial, the effects of sibeprenlimab on eGFR and long-term safety outcomes are not yet available. Furthermore, the Impact of Sibeprenlimab on Kidney Tissue study in patients with IgAN (NCT06740526) and the phase 2-3 open-label long-term extension study of sibeprenlimab (NCT05248659) are currently ongoing. We eagerly anticipate encouraging results from these studies.

Zigakibart Zigakibart (BION-1301) is a humanized IgG4 monoclonal antibody targeting APRIL. Similar to sibeprenlimab, it specifically binds and neutralizes APRIL without binding BAFF, thereby distinguishing it from the dual-target fusion protein atacicept. Based on the results of a phase 1/2 clinical trial (NCT03945318), zigakibart in 40 IgAN patients over 100 weeks showed a 60.4% reduction in 24-hour UPCR, with an annualized eGFR slope of +0.48 mL/min/1.73 m^2^ (95% CI −0.74–1.70). IgA, Gd-IgA1, and IgM fell by 74.3%, 74%, and 80%, respectively; IgG declined modestly (36%). Hematuria resolved in 82.4% of baseline-positive patients. No treatment-related discontinuations or deaths occurred (66). In addition, a preclinical study of zigakibart has validated, through both *in vitro* and *in vivo* experiments, that selective inhibition of APRIL represents a feasible disease-modifying strategy for the treatment of IgA nephropathy. These investigations have elucidated the pathophysiological mechanisms underlying the reductions in serum IgA and Gd-IgA1 levels, the attenuation of proteinuria, and the stabilization of renal function observed in patients with IgA nephropathy (67). Ongoing phase 3 follow-up studies, including NCT05852938 (67) and a multicenter open-label extension study (NCT06858319), are currently evaluating the effects of zigakibart on proteinuria and long-term renal function in adult patients with IgA nephropathy.

Telitacicept Telitacicept (RC18) is the first-in-class dual BAFF/APRIL-targeting TACI-Fc fusion protein, composed of the Fc fragment of human IgG1 fused to the extracellular domain of the transmembrane activator and calcium-modulating cyclophilin ligand interactor (TACI). Telitacicept has been approved for the indication of primary IgA nephropathy and represents a core therapeutic agent in precision-targeted immunotherapy for this disease (68). In a phase 3, multicenter, double-blind, randomized, placebo-controlled trial involving 318 patients, telitacicept 240 mg weekly for 39 weeks reduced 24-hour UPCR by 58.9% vs. 8.8% with placebo (relative difference −55.0%; *P* < 0.001). eGFR declined by only 1.0% with telitacicept vs. 7.7% with placebo; 6.3% vs. 27.0% had confirmed eGFR decline ≥30%. UPCR <0.8 was achieved in 61.0% vs. 19.5%. CD19+ B cells fell by 46.4% (vs. + 4.5%). Adverse events(AEs) were more frequent with telitacicept (89.3% vs. 78.6%), mainly injection-site reactions, but serious AEs were fewer (2.5% vs. 8.2%). No unexpected safety signals emerged (69). In a phase 2 study conducted in China involving 44 patients with IgA nephropathy (NCT04291781), telitacicept 240 mg reduced 24-hour proteinuria by 49% from baseline (*P* = 0.013 vs. placebo), whereas 160 mg reduced it by 25% (*P* = 0.389 vs. placebo) at 24 weeks. eGFR rose by +2.34 mL/min/1.73 m^2^ (240 mg) and +4.32 (160 mg) vs. −5.70 with placebo (*P* = 0.015 and 0.002). IgA fell ∼46%, IgG ∼25%, IgM ∼65%. No severe AEs or deaths occurred; injection-site reactions were more common with telitacicept (70). An exploratory biomarker analysis of plasma samples from the phase 2 trial further demonstrated that telitacicept reduced circulating Gd-IgA1 and IgA-containing immune complexes. At week 24, Gd-IgA1 decreased by 43.9% (160 mg group) and 50.4% (240 mg group) (71). Subsequent real-world studies of telitacicept have confirmed that its therapeutic effects are consistent with the efficacy trajectory established in the phase 2 trial (NCT04291781), and more pronounced renoprotective effects have been observed (72).

Atacicept In July 2026, atacicept received accelerated approval from the FDA for reducing proteinuria in adult patients with primary IgA nephropathy at risk of disease progression (73, 74). A phase 2 study demonstrated that atacicept had an acceptable safety profile, effectively reduced Gd-IgA1 levels, and showed potential for improving proteinuria and stabilizing renal function. However, the study was terminated prematurely due to slow subject enrollment (75). Subsequently, in a randomized, double-blind phase 3 clinical trial involving 376 patients (the ORIGIN 3 study, NCT04716231), atacicept 150 mg weekly for 36 weeks reduced UPCR by 45.7% vs. 6.8% with placebo (between-group difference 41.8 percentage points; *P* < 0.001). Gd-IgA1 fell by 68.3% vs. 2.9%; hematuria resolved in 81.0% vs. 20.7%; UACR(Urine Albumin-to-Creatinine Ratio) declined 47.3% vs. 8.8%. eGFR slope data are not yet reported (blinded per interim analysis). Most AEs were mild/moderate; serious AEs occurred in 0.5% (atacicept) vs. 5.1% (placebo); no deaths or opportunistic infections (76).

Povetacicept Povetacicept is an innovative dual-targeting BAFF/APRIL inhibitor. In an open-label, dose-escalation study of povetacicept (80 mg or 240 mg, subcutaneous every 4 weeks) for IgA nephropathy, at week 48, the 80-mg and 240-mg groups achieved 64% and 56% UPCR reductions, with eGFR changes of +3.3 and +3.2 mL/min/1.73 m^2^ respectively. Gd-IgA1 levels decreased by 77% in both cohorts. Clinical remission was attained in 53% (80 mg) and 34% (240 mg) of evaluable patients, while hematuria resolved in 90% and 94% of subjects. Povetacicept exhibited an acceptable safety profile in IgAN participants (30). An ongoing global, randomized, double-blind, placebo-controlled phase 3 pivotal trial has now completed enrollment, but the results have not yet been released (77). Across the current trials, povetacicept has demonstrated a favorable safety and tolerability profile. The vast majority of adverse events were mild to moderate in severity, with no drug-related serious adverse events or deaths observed, and no opportunistic infections reported (78, 79). The above findings support povetacicept as a promising disease-modifying therapy for IgAN.

### 4.2 Lupus nephritis

#### 4.2.1 BAFF/APRIL in the pathogenesis of lupus nephritis

Lupus nephritis (LN) is one of the common and severe complications of systemic lupus erythematosus (SLE). Its pathogenesis involves a loss of immune tolerance to endogenous nuclear material, leading to the persistent production of anti-DNA antibodies and related antinuclear antibodies, which in turn trigger a systemic autoimmune response and result in multi-organ damage. In the kidneys, persistent inflammation driven by immune dysregulation, combined with immune complex deposition, contributes to the development of LN. This process involves abnormal activation of B cells and T cells, as well as dysregulation of cytokine networks and the complement system (80, 81). Studies have shown that the BAFF/APRIL system is frequently overexpressed in patients with LN. Elevated serum BAFF/APRIL levels are positively correlated with disease activity (82), and other studies have confirmed that urinary BAFF/APRIL can serve as a biomarker reflecting local renal inflammation (83). BAFF and APRIL interact with receptors on the surfaces of B and T cells, inducing B cells to secrete pathogenic autoantibodies such as anti-dsDNA antibodies. These autoantibodies bind to autoantigens to form circulating immune complexes, which deposit in the glomerular capillary loops, activate the complement system, and cross-link Fc receptors, thereby inducing inflammatory cell infiltration, cytokine release, and renal tissue damage (81). Furthermore, numerous clinical studies have demonstrated that BAFF/APRIL-targeted therapies (such as belimumab and telitacicept) can effectively reduce proteinuria and mitigate kidney damage in patients with LN (9, 11). Therefore, BAFF/APRIL-based intervention strategies warrant close attention in the treatment of LN.

#### 4.2.2 BAFF/APRIL-targeted biologics

Belimumab Belimumab is a human IgG1*λ* monoclonal antibody that binds to and neutralizes the activity of soluble BAFF (84, 85). The FDA approved the anti-BAFF monoclonal antibody, belimumab, in 2020 for adult LN, and in 2022 for pediatric LN (86). BLISS-LN is a pivotal phase 3 clinical trial evaluating belimumab in the treatment of active lupus nephritis. A total of 448 subjects were randomized in a 1:1 ratio to receive intravenous belimumab (10 mg/kg once monthly) or placebo, both administered on top of standard immunosuppressive regimens comprising mycophenolate mofetil or cyclophosphamide. Results demonstrated that at week 104, belimumab significantly improved the primary efficacy renal response (43% vs. 32%; OR 1.6; *P* = 0.03) and complete renal response (30% vs. 20%; OR 1.7; *P* = 0.02). The partial renal response rate (ordinal analysis) was 18% vs. 17%, with no significant difference. Belimumab also reduced the risk of renal-related events or death (HR 0.51; *P* = 0.001). This confirmed that belimumab improves renal remission rates in patients with lupus nephritis, with a favorable tolerability profile (87). In another multicenter, randomized, open-label clinical trial involving 43 patients with refractory lupus nephritis, the safety and efficacy of rituximab plus cyclophosphamide followed by belimumab (RCB group) were compared with those of rituximab plus cyclophosphamide alone (RC group). The results showed that the addition of belimumab did not increase adverse events such as infections, and was associated with more sustained B-cell depletion and inhibition of autoreactive B-cell maturation. Among refractory patients with baseline heavy proteinuria (UPCR > 3), the remission rate at 48 weeks was 88% in the RCB group versus 43% in the RC group, suggesting a potential benefit in the subset with severe proteinuria. However, it should be noted that there was no statistically significant difference in renal remission rates between the two groups in the overall population (*p* = 0.452), indicating that although this combination regimen was safe and modulated B-cell immunity, it did not significantly improve clinical efficacy (88).

Telitacicept Regarding clinical trials of telitacicept in lupus nephritis, available evidence indicates that it significantly improves renal remission rates when added to standard therapy. Furthermore, telitacicept may exhibit a faster onset of action compared with belimumab and may reduce the requirement for glucocorticoids and immunosuppressive agents. However, these findings warrant further validation in larger-scale studies (89, 90). A phase 2, multicenter, randomized, double-blind, placebo-controlled study evaluating the efficacy and safety of telitacicept in lupus nephritis is currently recruiting participants (NCT05680480) (91). In addition, a retrospective multicenter cohort study comparing the efficacy and safety of telitacicept and belimumab in lupus nephritis (NCT07031674) has been completed, although the results have not yet been published (92). Telitacicept is a TACI-Fc fusion protein that simultaneously targets both BAFF and APRIL, two key cytokines, which may underlie its enhanced efficacy compared with belimumab, which targets BAFF alone. Collectively, the available evidence supports telitacicept as a promising therapeutic option for lupus nephritis, particularly in the pursuit of rapid and deep remission.

Atacicept Atacicept (Trutakna) is a recombinant soluble fusion protein that simultaneously targets both BAFF and APRIL (5). A phase 2/3, randomized, double-blind, placebo-controlled study was designed to evaluate the safety and efficacy of atacicept in combination with mycophenolate mofetil in subjects with lupus nephritis. However, after enrolling only six patients, the atacicept group (*n* = 4) experienced marked and rapid declines in serum IgG levels, with three patients falling below the predefined discontinuation threshold of 3 g/L, accompanied by serious infections, including two cases of severe pneumonia. Of note, during the screening period prior to atacicept administration (when patients were receiving only MMF and high-dose corticosteroids), IgG levels had already decreased substantially, suggesting that the background immunosuppressive regimen itself carried a significant risk. Consequently, the trial was terminated early, precluding any assessment of efficacy (93). These findings suggest that although atacicept holds theoretical therapeutic promise in lupus nephritis, its clinical utility remains constrained by significant safety concerns, particularly serious infections, as observed in clinical trials. Further rigorous studies are therefore warranted to define its role in the management of lupus nephritis.

### 4.3 Primary membranous nephropathy

#### 4.3.1 BAFF/APRIL in the pathogenesis of primary membranous nephropathy

Primary membranous nephropathy (pMN) is an autoimmune glomerular disease characterized by circulating autoantibodies, predominantly against phospholipase A2 receptor (PLA2R) and thrombospondin type-1 domain-containing 7A (THSD7A) (94–96). Circulating anti-PLA2R autoantibodies bind to their target antigens on glomerular podocytes, forming immune complexes that deposit and activate complement, thereby triggering local inflammation and tissue injury, which in turn drive the onset and progression of membranous nephropathy (97). In addition, clinical studies have demonstrated that serum BAFF and APRIL levels are significantly elevated in patients with pMN and are closely correlated with anti-PLA2R antibody titers and the severity of proteinuria (98). BAFF and APRIL provide critical survival signals to pathogenic B cells that secrete anti-PLA2R autoantibodies, inhibiting apoptosis and enabling the persistent activation and persistence of pathogenic B cell clones. Among these, APRIL sustains the survival of long-lived plasma cells through binding to the BCMA receptor, thereby ensuring the continuous production of high-titer anti-PLA2R antibodies. Some studies have also suggested that BAFF may be directly involved in podocyte injury, further aggravating glomerular lesions (99, 100). Clinical trials of the dual BAFF/APRIL inhibitor povetacicept have shown that, following treatment, pMN patients achieved a 83% reduction in anti-PLA2R antibody levels and an 82% reduction in proteinuria, with a markedly improved clinical remission rate. These findings provide compelling evidence that the BAFF/APRIL signaling pathway plays a central regulatory role in the pathogenesis of pMN (101).

#### 4.3.2 BAFF/APRIL-targeted biologics

Belimumab In a 2-year mechanistic study of belimumab (NCT01610492), 14 patients with PLA2R Ab-positive pMN received belimumab monotherapy (10 mg/kg intravenous every 4 weeks, up to 2 years). At Week 28, anti-PLA2R antibodies fell by 73% (geometric mean from 174 to 46 RU/mL), with reductions significant by Week 12 (46%; *P* = 0.02). Proteinuria (UPCR) declined by 31% at Week 28 (724–498 mg/mmol) and reached a significant 56% reduction at Week 36 (*P* = 0.047), continuing to 86% by Week 104 (130 mg/mmol). Overall, 64% of patients (9/14) achieved partial or complete remission; complete antibody response occurred in 10/14. The results demonstrated that belimumab reduced anti-PLA2R antibody levels in patients, which was subsequently associated with a decrease in proteinuria, thereby providing an important foundation for inducing disease remission (29). Currently, another phase 2 study evaluating belimumab in combination with rituximab versus rituximab monotherapy for primary membranous nephropathy (NCT03949855) is actively recruiting participants (102).

Telitacicept/Atacicept At present, biologic therapy with telitacicept or atacicept for membranous nephropathy remains in the exploratory phase. Two relevant phase 2 trials merit attention: one evaluating telitacicept in primary membranous nephropathy (NCT06614985) (103), which has yet to begin recruitment, and another assessing atacicept in multiple autoimmune glomerular diseases (NCT06983028) (104), which is actively enrolling participants. Although telitacicept and atacicept have demonstrated established efficacy in IgA nephropathy and systemic lupus erythematosus, their therapeutic value in membranous nephropathy still awaits clinical validation.

Povetacicept Currently, no approved therapies are available for pMN. Povetacicept targets the pathogenic origin of pMN by rapidly and substantially reducing the levels of disease-causing autoantibodies, specifically anti-PLA2R antibodies. In an open-label phase 1/2 RUBY-3 study (NCT05732402), 10 patients with pMN received povetacicept 80 mg subcutaneously every 4 weeks. Early decline in aPLA2R at week 12 (73%), continued at week 48 (83%), with 100% achieving immunological remission (<14 RU/mL). Proteinuria (UPCR) decreased by 82% (from 3.8 to 0.7 g/g); in the nephrotic-range subset (UPCR ≥ 3.5 g/g, *n* = 6), UPCR fell by 85% and eGFR improved by +2.2 mL/min/1.73 m^2^. Overall remission was 100% (40% complete). Of note, one case of asymptomatic severe hypogammaglobulinemia (IgG < 150 mg/dL) occurred, leading to protocol-mandated treatment discontinuation. However, no associated infections were reported (30). The above data indicate that povetacicept, as a dual BAFF/APRIL-targeting inhibitor, achieves profound and durable antibody depletion and clinical remission through upstream modulation of B-cell and plasma cell function, thereby offering a highly promising disease-modifying therapeutic strategy for pMN. A phase 3 clinical trial (NCT07204275) is currently ongoing.

### 4.4 Other autoimmune kidney diseases

Despite encouraging progress with BAFF/APRIL inhibitors in IgA nephropathy, lupus nephritis, and primary membranous nephropathy, their therapeutic value in other autoimmune kidney diseases (including anti-GBM disease and ANCA-associated nephritis) has not been established (105). Evidence supporting BAFF/APRIL-targeted therapy in these conditions remains insufficient, and its application should currently be considered strictly exploratory.

### 4.5 Cross-trial analysis within standardized time frames

Because individual trials reported endpoints at different time points (24, 36, 48, and 104 weeks), all cross-trial comparisons in this section are organized according to three standardized time frames: short-term (≤24–28 weeks), mid-term (36–52 weeks), and long-term (≥100 weeks). Direct numerical comparisons across different time frames are not appropriate, given differences in treatment exposure, baseline characteristics, and background immunosuppression. Detailed trial-level data are provided in Table 2.

**Table 2:** Efficacy endpoints of completed and interim-reported clinical trials of BAFF/APRIL-targeted biologics for IgAN, LN, and pMN, by standardized time frame.

Time frame	Biologics	Disease	Endpoint time	Proteinuria reduction	eGFR change	Immunological endpoints
short-term (≤24–28 weeks)	Sibeprenlimab NCT03719443	IgAN	16–24 weeks	Not reported	Not reported	IgA −57.2%, IgG −33.6%, IgM −67.2%, and Gd-IgA1 −71.6% at Week 12.
	Telitacicept NCT04291781	IgAN	24 weeks	24-h proteinuria −49%(240 mg) and −25%(160 mg).	eGFR: +2.34 (240 mg) and +4.32 (160 mg) mL/min/1.73 m^2^ vs. –5.70 (placebo)	IgA fell ∼46%, IgG ∼25%, IgM ∼65%. Gd-IgA1 −43.9%/−50.4% (160/240 mg).
	Atacicept NCT02808429	IgAN	24 weeks	24-h UPCR median change from baseline: placebo +24%; atacicept 25 mg −24%; atacicept 75 mg −25%.	Median change from baseline in eGFR: placebo −7%; atacicept 25 mg −7%; atacicept 75 mg −4%.	IgA: placebo +4%; atacicept 25 mg −13%; atacicept 75 mg −52%. IgG: −8%; −10%; −31%. IgM: −4%; −38%; −69%. Gd-IgA1: +2%; −25%; −60%.
	Belimumab NCT01610492	pMN	28 weeks	UPCR: −31%(geomean 724→498 mg/mmol).	Stable eGFR overall.	PLA2R:-Ab: 73% reduction from baseline; geomean decreased from 168 RU/mL to 46 RU/mL).
mid-term (36–52 weeks)	Sibeprenlimab NCT04287985	IgAN	12 months	24-h UPCR: −47.2%/58.8%/62.0% (2/4/8 mg/kg) vs. −20.0% placebo.	Annualized eGFR slope: –4.1, +0.1, –0.8 mL/min/1.73 m^2^/year vs. –5.9 placebo.	Gd-IgA1 and IgA reduced by ∼65%, IgG by ∼35%, and IgM by ∼75%.
	Sibeprenlimab NCT05248646	IgAN	9–12 months	24-h UPCR:−50.2% (sibeprenlimab) vs. +2.1% (placebo) at 9 months; −56.6% vs. −5.1% at 12 months.	Not reported	Gd-IgA1 −67.1%, APRIL −95.8%, IgA −68.8%, IgG −35.0%, IgM −74.5%.
	Telitacicept NCT05799287	IgAN	39 weeks	24-h UPCR: −58.9%(telitacicept) vs. −8.8%(placebo); Proteinuria response (UPCR <0.8): 61.0% vs. 19.5% with placebo.	eGFR stable (telitacicept) vs. ↓(placebo). Confirmed ≥30% eGFR: −6.3% vs. −27.0%.	CD19+ B-cell counts decreased by 46.4% with telitacicept (vs. +4.5% with placebo). Serum IgA fell by 60.6%.
	Atacicept NCT04716231	IgAN	36 weeks	24-h UPCR: −45.7% (atacicept) vs. −6.8% (placebo); UACR −47.3% vs. −8.8% (placebo).	eGFR slope results not reported in this interim analysis.	Galactose-deficient IgA1 decreased by 68.3% with atacicept vs. 2.9% with placebo.
	Povetacicept NCT05732402	IgAN	48 weeks	24-h UPCR: −64% (80 mg) vs. −56%(240 mg)	.Mean eGFR change: +3.3 mL/min/1.73 m^2^ (80 mg) and +3.2 mL/min/1.73 m^2^ (240 mg); annualized eGFR slope not reported.	Early Gd-IgA1 reductions at Week 12: –57% (80 mg), –59% (240 mg); deepened to –77% in both cohorts by Week 48.
	Belimumab NCT02260934	LN	48 weeks	Complete renal response: 38% (RCB) vs. 32% (RC). Partial renal response: 14% vs. 9%. Overall renal response (complete + partial): 52% vs. 41%(*p* = 0.452).		ANA + naive B cells decreased from baseline in 8/9 RCB participants vs. increased in 5/7 RC participants (*p* = 0.0349).
	Atacicept NCT00573157	LN	52weeks	Not reported	Not reported	3 patients: IgG < 3 g/L (protocol discontinuation threshold)
	Povetacicept NCT05732402	pMN	48 weeks	24-h UPCR: −82% (3.8→0.7 g/g). Baseline UPCR ≥3.5 g/g (*n* = 6): −85%. Overall clinical remission: 100% (5/5);	Stable eGFR: mean change –0.3 mL/min/1.73 m^2^. In the subset with baseline UPCR ≥3.5 g/g: eGFR change +2.2 mL/min/1.73 m^2^.	aPLA2R −73% (W12), −83% (W48; 98% in nephrotic subset). Immunologic remission (aPLA2R < 14 RU/mL): 100% at W48.
long-term (≥100 weeks)	Blisibimod NCT02062684	IgAN	168 weeks	24-h UTP: Slight fluctuation initially, marked decline later (Blisibimod); Stable early, continuous increase after mid-term (placebo).	Annualized eGFR slope(mL/min/1.73 m^2^/year): +6.2 with blisibimod vs. –4.8 with placebo.	Serum immunoglobulins (IgA, IgG, IgM) showed marked reductions throughout treatment.
	Zigakibart NCT03945318	IgAN	100 weeks	24-h UPCR: −60.4%(Zigakibart).	Annualized eGFR slope: +0.48 mL/min/1.73 m^2^/year.	IgA −74.3% (W 100), Gd-IgA1 −74% (W 76), IgM −80% (Week 100), IgG −36%.
	Belimumab NCT01639339	LN	104 weeks	Primary efficacy renal response: 43% (belimumab) vs. 32% (placebo). Complete renal response: 30% vs. 20%. Partial renal response: 18% vs. 17%.		Not reported

UPCR, urine protein-to-creatinine ratio; eGFR, estimated glomerular filtration rate; W, week; UP, urine protein; TEAE, treatment-emergent adverse event; AEs, adverse events; SAEs, serious adverse events; Unit conversion, UPCR 1 mg/mmol ≈ 0.00884 g/g.

#### 4.5.1 Short-term outcomes (≤24–28 weeks)

In the short-term layer, three agents reported comparable 24-week proteinuria endpoints in IgAN. Telitacicept 240 mg reduced 24-h proteinuria by 49% versus placebo (LS mean difference −0.88 g/day; *P* = 0.013), accompanied by an eGFR increase of +2.34 mL/min/1.73 m^2^. Atacicept showed a dose-dependent reduction in 24-h UPCR (25 mg: −24%; 75 mg: −25%) with a smaller eGFR decline than placebo (−4% to −7% vs. −7%), together with marked reductions in IgA, IgM, and Gd-IgA1 at the 75 mg dose. In pMN, belimumab monotherapy reduced UPCR by 31% at 28 weeks and anti-PLA2R antibodies by 73%, with stable eGFR. Although these short-term results consistently demonstrate early proteinuria reduction and immunological suppression, the trials differ in disease, baseline UPCR, and background therapy; therefore, the magnitude of reduction should be interpreted as indicative rather than directly comparable.

#### 4.5.2 Mid-term outcomes (36–52 weeks)

In the mid-term layer, several agents can be compared within the same time frame:

IgAN: Sibeprenlimab (12 months) reduced 24-h UPCR by 47.2–62.0% versus 20.0% with placebo, with an annualized eGFR slope of −4.1 to +0.1 mL/min/1.73 m^2^/year versus −5.9 with placebo. The phase 3 interim analysis reported a 56.6% UPCR reduction at 12 months and near-complete APRIL suppression, although eGFR slope data remain blinded. Telitacicept (39 weeks) reduced UPCR by 58.9% versus 8.8% with placebo, with stable eGFR (−1.0% vs. −7.7%) and a 46.4% reduction in CD19+ B cells. Atacicept (36 weeks) reduced UPCR by 45.7% versus 6.8% with placebo and lowered Gd-IgA1 by 68.3%, but eGFR slope data were not reported. Povetacicept (48 weeks) achieved 56%–64% UPCR reductions with eGFR increases of +3.2 to +3.3 mL/min/1.73 m^2%^ and 77% Gd-IgA1 reduction. Blisibimod failed to meet its primary endpoint at Week 48, although extended treatment suggested eGFR preservation (+6.2 vs. −4.8 mL/min/1.73 m^2^/year).

LN: The RCB trial (48 weeks) reported an overall renal response rate of 52% versus 41% (*p* = 0.452), with a pronounced benefit in the subgroup with baseline UPCR >3 (88% vs. 43%). The absence of statistical significance in the overall population likely reflects the small sample size and the refractory disease profile.

pMN: Povetacicept (48 weeks) reduced UPCR by 85% in the nephrotic-range subset, with a mean eGFR increase of +2.2 mL/min/1.73 m^2^ and overall remission was 100%.

Within this layer, telitacicept, sibeprenlimab, atacicept, and povetacicept all achieved 45%–64% UPCR reductions, but the differences in baseline eGFR (65.3–79.4 mL/min/1.73 m^2^), baseline UPCR (1.23–3.8 g/g), race composition, and background therapy preclude direct numerical comparison. The apparent superiority of povetacicept in pMN, for example, may partly reflect its lower baseline UPCR and permitted calcineurin inhibitor background.

#### 4.5.3 Long-term outcomes (≥100 weeks)

In the long-term layer, belimumab (BLISS-LN, 104 weeks) improved the primary efficacy renal response (43% vs. 32%; OR 1.6; *P* = 0.03) and complete renal response (30% vs. 20%; OR 1.7; *P* = 0.02), and reduced the risk of renal-related events or death (HR 0.51; *P* = 0.001). Belimumab monotherapy in pMN (104 weeks) reduced proteinuria by 86%. Zigakibart (100 weeks) reduced 24-h UPCR by 60.4% with an annualized eGFR slope of +0.48 mL/min/1.73 m^2^, and blisibimod (168 weeks) showed a mean 24-h UPCR decline from 2.02 to 1.18 g alongside an annualized eGFR improvement of +6.2 mL/min/1.73 m^2^/year. These long-term data should not be compared directly with short- or mid-term results from other trials.

#### 4.5.4 Cross-time-frame trends (qualitative only)

Across time frames, a consistent pattern emerges: proteinuria reduction is observed early (short-term), while eGFR stabilization or improvement becomes apparent only in mid- and long-term follow-up. However, because of differences in baseline eGFR, UPCR, race composition, and background immunosuppression, these trends are descriptive rather than directly comparative. In particular, the marked contrast between IgAN trials (supportive care background) and LN trials (MMF/CYC plus glucocorticoids) means that efficacy and safety signals should be interpreted within their respective background-therapy contexts.

## 5 A working translational hypothesis on the differential contribution of BAFF and APRIL in IgAN, LN, and pMN

In summarizing the clinical trial data of BAFF/APRIL-targeting biologics in autoimmune kidney disease, we observed substantial heterogeneity in clinical responses, prompting us to formulate a working translational hypothesis: the pathogenic contributions of BAFF and APRIL may differ across various kidney diseases. However, this hypothesis is derived from indirect, cross-trial comparisons that are confounded by differences in patient cohorts, study durations, endpoints, and background regimens, and it currently lacks validation from direct head-to-head mechanistic or clinical trials. Therefore, the following framework should be viewed as a working translational hypothesis rather than an established clinical fact. With this important caveat, we propose that: BAFF/APRIL dual inhibitors and APRIL-specific agents have demonstrated definitive efficacy in IgAN, whereas BAFF blockade appears preferentially beneficial in LN. Notably, emerging evidence in pMN has been interpreted to suggest a BAFF-dominant role, although this interpretation remains preliminary and requires further validation.

1. IgAN: IgA nephropathy can be regarded as a paradigmatic example in which APRIL-targeted therapy (e.g., sibeprenlimab, zigakibart) has achieved substantial clinical efficacy. Although the failure of the selective BAFF inhibitor blisibimod to meet its primary endpoint in the BRIGHT-SC trial is sometimes cited in support of this view, it must be acknowledged that this trial had methodological limitations in sample size and design. Mechanistically, APRIL, acting through TACI and BCMA, sustains mucosal plasma cell survival and drives IgA class switching via TACI. BAFF, by contrast, predominantly engages BAFF-R to maintain transitional and naïve B cell homeostasis (14, 15, 57). Genome-wide association studies (GWAS) have definitively identified TNFSF13, the gene encoding APRIL, as a risk locus for IgAN (106, 107). There is a stronger clinical correlation between APRIL induction and disease activity. In murine experiments, treatment with VIS649 (a humanized anti-APRIL IgG2*κ* antibody) reduced serum IgA by up to 70% (108). By contrast, anti-BAFF antibody (belimumab) lowered IgA by only approximately 16% (109), suggesting that APRIL plays a more critical role in regulating pathogenic IgA. In IgAN, the APRIL–TACI axis is the dominant driver of Gd-IgA1 production, but BAFF can still amplify the pool of autoreactive B cells and, through shared TACI signaling, contribute to IgA production. Because BAFF and APRIL share TACI and can partially compensate for one another, selective BAFF blockade may leave the APRIL–TACI axis intact, whereas selective APRIL blockade may still permit BAFF-R–dependent survival of autoreactive B cells. Simultaneous neutralization of both ligands is therefore expected to suppress the pathogenic B-cell/plasma-cell axis more completely than blockade of either ligand alone. This provides a coherent explanation for why dual BAFF/APRIL inhibitors (telitacicept, atacicept, povetacicept) achieve robust clinical responses in IgAN, whereas selective BAFF blockade with blisibimod did not meet its primary endpoint. Taken together, these observations indicate that APRIL appears to be the dominant but not the sole driver in IgAN.
2. LN: The therapeutic success of belimumab, a selective BAFF inhibitor, inLN has established BAFF as a key driver of this disease. The pathogenesis of LN is fundamentally characterized by loss of B-cell tolerance, which permits the survival and expansion of autoreactive B-cell clones and the subsequent production of pathogenic antinuclear antibodies, including anti-dsDNA. The survival of these autoreactive B cells during their transitional and mature stages is highly dependent on BAFF-R–mediated signaling, wherein BAFF delivers antiapoptotic signals through the NF-*κ*B and PI3K-Akt pathways, serving as an essential checkpoint (110, 111). BAFF-transgenic mice spontaneously develop lupus-like autoimmune disease (112, 113), and patients with LN exhibit significantly elevated serum BAFF bioactivity, further supporting its pathogenic role (114). The BLISS-LN trial demonstrated that the addition of belimumab to standard therapy significantly improved the renal response rate (87). On the other hand, APRIL has also been found to be highly expressed in renal tissues of patients with proliferative LN, and proteinuria has been shown to correlate significantly with elevated APRIL levels in these patients, suggesting a role for APRIL in exacerbating autoimmune inflammation and promoting macrophage infiltration (83, 115, 116). Accordingly, combined inhibition of APRIL on top of BAFF blockade may more thoroughly deplete the pathogenic B-cell pool, potentially improving remission rates and reducing relapse risk in LN—a strategy currently being evaluated in ongoing clinical studies.
3. pMN: pMN represents an emerging area of investigation for BAFF/APRIL-targeted therapy. Available clinical data suggest that the association between BAFF and the pathogenesis of membranous nephropathy is better established and more definitive than that between APRIL and this disease (98, 117), leading to the proposal that BAFF may play a more central pathogenic role. At the mechanistic level, injured podocytes may contribute to shaping the renal local BAFF-mediated immune microenvironment. On the one hand, podocytes have been shown to express BAFF, although the evidence for this in primary membranous nephropathy remains limited (118, 119). On the other hand, damaged podocytes can release a variety of chemokines and cytokines that recruit and activate myeloid immune cells within the glomerulus. These myeloid cells, in turn, can secrete BAFF, thereby perpetuating local autoimmune injury in the kidney. However, this remains to be validated in further *in vivo* studies (120–122). Although the above findings suggest that BAFF may contribute to the pathogenesis of pMN, it must be emphasized that the current rationale for regarding pMN as a “BAFF-dominant” disease remains preliminary and speculative. Conflicting mechanistic evidence indicates that APRIL also plays a key role in this disease. Specifically, Long-lived plasma cells residing in the bone marrow produce anti-PLA2R antibodies and express high levels of BCMA, making them heavily dependent on APRIL (25, 26, 99, 100). Furthermore, the marked clinical efficacy of povetacicept in pMN most likely reflects its potent dual inhibition of both BAFF and APRIL rather than BAFF inhibition alone, further suggesting that a contribution of APRIL cannot be excluded (101). At present, whether APRIL independently contributes to pMN pathogenesis remains unclear. The relative contribution of BAFF and APRIL in pMN remains unclear and requires further investigation.

However, these conclusions should be interpreted as a working hypothesis derived from indirect preclinical, genetic, and fragmented clinical observations, and thus must be interpreted with caution. Many dedicated selectivetarget clinical studies are still lacking: direct headtohead selective BAFF versus selective APRILintervention trials are limited for pMN and partially for LN. Observations from indirect comparisons across distinct trials cannot definitively prove one molecule is pathologically “dominant” over another in a given disease. Formal validation of this hypothesis will require prospective mechanistic studies and dedicated head-to-head clinical trials. Until such evidence emerges, this framework should be viewed as a conceptual guide for hypothesis generation rather than a definitive therapeutic algorithm for biologic selection.

## 6 Confounding background therapies and safety profiles

### 6.1 Impact of background immunosuppression on efficacy and safety interpretation

A critical confounder in cross-trial comparisons of BAFF/APRIL-targeted biologics is the marked heterogeneity in background immunosuppressive regimens across disease entities. This difference has two important consequences. First, it directly affects the interpretation of efficacy. IgAN trials restricted systemic immunosuppressants and focused on RAS/SGLT2 inhibitors. Systemic immunosuppressants were explicitly prohibited (e.g., NCT04287985, NCT05248646). The observed proteinuria reduction can be attributed more directly to BAFF/APRIL blockade, because background therapy contributes little to immunological suppression. Conversely, In lupus nephritis, belimumab was studied as an add-on to high-dose steroids and MMF/CYC (BLISS-LN), which may mask the full therapeutic effect of the biologic and compress the between-group difference. Similarly, for pMN, the RUBY-3 study of povetacicept permitted stable calcineurin inhibitor therapy as an optional background treatment, whereas the belimumab mechanistic study excluded immunosuppressive agents. This difference in background therapy may partly explain the higher remission rate observed with povetacicept, although dual BAFF/APRIL inhibition by the biologic agent may also have contributed. Future trials should standardize background immunosuppressive therapy or perform subgroup analyses stratified by background treatment to clarify the independent contribution of biologic agents. Second, background immunosuppression modifies the safety profile. In the atacicept LN trial (NCT00573157), patients receiving MMF plus high-dose glucocorticoids experienced a marked and rapid decline in serum IgG, with three of four patients falling below the predefined discontinuation threshold of 3 g/L, accompanied by severe infections including two cases of pneumonia. Notably, IgG levels had already declined substantially during the screening period, before atacicept administration, suggesting that the background regimen itself contributed to the hypogammaglobulinemia risk. In contrast, IgAN trials with minimal background immunosuppression reported fewer serious infectious events. This contrast underscores that the safety of BAFF/APRIL blockade cannot be evaluated in isolation from the concomitant immunosuppressive context.

### 6.2 Safety profile: hypogammaglobulinemia, infection risk, and mitigation strategies

Given that BAFF and APRIL are essential for B-cell survival, plasma cell maintenance, and immunoglobulin production, it is not unexpected that their blockade can lead to hypogammaglobulinemia and an associated increase in infection risk. This class effect has emerged as one of the most important safety considerations for BAFF/APRIL-targeted biologics, particularly for dual inhibitors and APRIL-selective agents.

Hypogammaglobulinemia. The most instructive example remains the phase 2/3 atacicept trial in LN (NCT00573157), which was terminated prematurely after only six patients were enrolled. In the atacicept group (*n* = 4), serum IgG declined rapidly, with three patients falling below the predefined discontinuation threshold of 3 g/L, accompanied by serious infections including two cases of severe pneumonia. Importantly, IgG levels had already decreased during the screening period, when patients were receiving only MMF and high-dose corticosteroids, indicating that the background immunosuppressive regimen itself contributed substantially to the risk. In IgAN trials, APRIL-selective agents such as sibeprenlimab and zigakibart produced substantial reductions in serum IgA, Gd-IgA1, and IgM, with only modest reductions in IgG (approximately 35% for sibeprenlimab and 36% for zigakibart), and no treatment-related discontinuations or deaths were reported. Similarly, in the pMN RUBY-3 study of povetacicept, one case of asymptomatic severe hypogammaglobulinemia (IgG <150 mg/dL) occurred and led to protocol-mandated treatment discontinuation, but no associated infections were observed. These observations suggest that the depth and duration of IgG suppression, rather than the mere occurrence of hypogammaglobulinemia, determine infectious risk.

Infection risk. Serious infections have been reported across BAFF/APRIL-targeted programs, but their incidence varies with the degree of immunoglobulin depletion and the intensity of background immunosuppression. In BLISS-LN, the overall safety profile of belimumab was consistent with previous trials, with infections being the most common adverse event, but no unexpected safety signals emerged. In the atacicept LN trial, however, the combination of MMF, high-dose glucocorticoids, and dual BAFF/APRIL blockade produced life-threatening infections, highlighting the potential for cumulative immunosuppression.

Mitigation strategies. Based on the available evidence, several practical measures should be considered for clinical use and future trial design:

1. Baseline and serial IgG monitoring. Serum IgG should be measured before initiation and at regular intervals during treatment. A threshold of 3 g/L has been used as a discontinuation criterion in atacicept trials, whereas a threshold of 150 mg/dL (severe hypogammaglobulinemia) was used in the povetacicept pMN study. Trials should predefine IgG monitoring intervals and discontinuation thresholds.
2. Risk stratification by background therapy. Patients receiving concomitant MMF, CYC, or high-dose glucocorticoids may require more frequent IgG monitoring, because the background regimen itself can contribute to hypogammaglobulinemia.
3. Infection prophylaxis. In patients with sustained IgG decline or a history of recurrent infections, prophylaxis against encapsulated organisms (e.g., pneumococcal and meningococcal vaccination, and in selected cases antibiotic prophylaxis) should be considered. Live vaccines should be avoided during active B-cell depletion.
4. Long-term extension studies. Real-world data and long-term extension studies are needed to define the durability of IgG suppression, the reversibility of hypogammaglobulinemia after treatment discontinuation, and the long-term infection risk.

In summary, while BAFF/APRIL-targeted biologics offer a mechanism-based therapeutic paradigm for autoimmune nephropathy, their safety profile—particularly the risk of hypogammaglobulinemia and infection—must be carefully managed through structured IgG monitoring, risk stratification by background immunosuppression, and predefined mitigation strategies.

## 7 Discussion

In this review, we systematically delineate the molecular mechanisms by which the BAFF/APRIL system drives aberrant activation of autoreactive B cells, promotes pathogenic plasma cell differentiation, and sustains the continuous production of pathogenic autoantibodies-including anti-dsDNA, anti-PLA2R, and Gd-IgA1-which serve as the core drivers of LN, pMN, and IgAN, respectively. Beyond its effects on B cells, this system exerts coordinated regulatory influences on T-cell subsets, promoting the differentiation of Tfh and Th17 cells while suppressing Treg function. Furthermore, it modulates innate immune cells such as dendritic cells and enhances the secretion of proinflammatory cytokines, thereby amplifying the autoimmune inflammatory cascade. Collectively, these actions underscore the multilayered, network-based immunomodulatory properties of this pathway. By orchestrating the interplay among B cells, T cells, and other immune cells, the BAFF/APRIL system bridges innate and adaptive immunity and serves as a critical nexus in the pathogenesis of autoimmune kidney diseases.

Clinical translation of BAFF/APRIL-targeting biologics has advanced considerably, with notable success in IgAN and LN. Sibeprenlimab, telitacicept, and atacicept are now approved for IgAN, and belimumab for LN, signaling a shift from nonspecific immunosuppression to mechanism-guided precision therapy. Unlike conventional immunosuppressants, these agents effectively reduce proteinuria and preserve renal function while directly targeting B-cell survival and autoantibody pathways, offering a biologically rational approach that may spare normal immune defenses. In pMN, however, this strategy remains under clinical investigation. Although anti-PLA2R antibody-mediated podocyte injury shares B-cell dependence with IgAN and LN, it remains unclear whether pMN autoantibody production is similarly BAFF/APRIL-dependent or whether pathway inhibition can clear established plasma cells. High-quality randomized controlled trials are needed to establish whether this approach can become standard of care for pMN and extend benefit to more patients with autoimmune kidney disease.

## 8 Challenges and future perspectives

Despite these advances, several challenges remain. First, the long-term safety profile of these biologics, particularly with respect to infection risk and immunoglobulin depletion, requires further elucidation. The premature termination of the atacicept trial in lupus nephritis due to severe infections and a marked decline in IgG levels serves as a cautionary example. Second, the optimal timing, treatment duration, and patient stratification strategies have yet to be established. Although biomarkers such as serum BAFF/APRIL levels, anti-PLA2R titers, or Gd-IgA1 may guide individualized therapy, their predictive value requires prospective validation. Third, the absence of head-to-head comparative trials among different biologics limits evidence-based decision-making. Ongoing studies, such as the comparison of telitacicept and belimumab in LN, are expected to provide valuable insights. In addition, the role of BAFF/APRIL in pMN represents an emerging area of research. Early data for povetacicept have shown encouraging reductions in both anti-PLA2R antibody levels and proteinuria. However, confirmatory phase III trial results are eagerly awaited. Exploration of combination regimens, such as belimumab plus rituximab, may offer synergistic benefits, although the potential cumulative immunosuppressive effects warrant careful consideration.

From a translational medicine perspective, future directions for BAFF/APRIL-targeted therapy may leverage biomarker-driven patient selection and dose optimization. Additionally, combining BAFF/APRIL inhibitors with other immunomodulators, including complement blockers and anti-CD38 antibodies, holds promise for achieving deeper and more sustained responses. Equally important, real-world data and long-term extension studies are critical to assess durable kidney function protection and safety over time.

In summary, the BAFF/APRIL system represents a validated and clinically actionable pathway in autoimmune kidney diseases. Although current biologics have demonstrated clear efficacy, future research will need to address outstanding questions regarding safety, durability of response, and patient heterogeneity. Ongoing exploration of its immunobiology, together with robust clinical research, will further optimize treatment approaches for these disabling renal disorders.
