# Supplementary material for: The BAFF/APRIL system in autoimmune nephropathy: from pathogenesis to targeted biologic therapies
Source: Front Med (Lausanne). 2026 Oct 8;13:1965501. doi: 10.3389/fmed.2026.1965501 (PMC13687708; doi:10.3389/fmed.2026.1965501)
Supplement: Supplementary file 1 [file Table1.docx]

Supplementary Material

**Supplementary Table 1.** Comparative summary of completed and interim‑reported clinical trials of BAFF/APRIL‑targeted biologics for IgAN, LN, and pMN.

| Biologics | Disease | Key inclusion/exclusion criteria | Baseline characteristics | Intervention regimen | Primary endpoint | Key secondary endpoints |
| --- | --- | --- | --- | --- | --- | --- |
| Blisibimod  NCT02062684 | IgAN | Inclusion Criteria:   1. Age 18–65 years;   2. Historical kidney biopsy–proven IgA nephropathy (Oxford classification);  3. Persistent proteinuria: 1–6 g/24 hours despite ACEi and/or ARB therapy;  4. eGFR > 30 mL/min/1.73 m²;  5. On stable, optimal ACEi and/or ARB for ≥90 days before randomization (continued as background therapy).  Exclusion Criteria:  1. Advanced glomerular or tubulointerstitial scarring;  2. Rapidly progressive glomerulonephritis. | 1. Mean age: Not specified (range 18–65)  2. Region: Asia (74%), Europe (26%);  3. All patients: Taking ARB;  4. ACEi+ARB use: 52%;  5. Mean time since diagnosis: 2.2 years;  6. Mean 24h urine protein: 2.14 g/day;  7. Mean eGFR: 68.25 mL/min/1.73 m²;  8. Mean Oxford Classification Derived Scor: 6.2. | Agent: Blisibimod;  Dose: 300 mg/week for 8 weeks, then 200 mg/week thereafter;  Comparator: Matching placebo;  Duration: At least 24 weeks; extension up to 104 weeks;  Background: Stable ACEi/ARB continued throughout;  Rescue therapy: Corticosteroids/immunosuppressives allowed after randomization if needed. | Number of patients achieving partial or complete response in urinary protein excretion  Response Definitions:  Partial response: Proteinuria ≤1 g/24h.  Complete response:  1. Baseline proteinuria 1–2 g/24h: proteinuria ≤1.0 g/24h AND ≥50% reduction from baseline at 2 consecutive visits;  2. Baseline proteinuria >2 g/24h: proteinuria ≤1.0 g/24h OR ≥50% reduction from baseline at 2 consecutive visits. | 1. Mean effects on proteinuria;  2. Pharmacodynamics: circulating B cells, B-cell subpopulations, serum immunoglobulins. |
| Sibeprenlimab  NCT03719443 | IgAN | Inclusion Criteria:  1. Healthy adults aged 18–55 years;  2. BMI 18–32 kg/m²;  3. Judged healthy by investigator based on medical evaluation and labs;  4. Serum IgG > 750 mg/dL, IgM > 55 mg/dL, IgA > 80 mg/dL;  5. Japanese or non-Japanese descent (based on grandparents’ origin).  Exclusion Criteria:  1. History/serious medical or psychiatric condition;  2. Proteinuria, CKD, or immunosuppressed status;  3. Antibody/biological therapy within 30 days or 5 half-lives;  4. Severe allergy/hypersensitivity to tetanus/diphtheria vaccine;  5. Tetanus vaccination within past 5 years;  6. Known hypoglobulinemia disorder;  7. Latent infection, infection requiring treatment, or vaccination within 30 days;  8. Use of systemic immunosuppressants/immunomodulators;  9. Positive urine drug, alcohol, or cotinine test. | 1. Age: 32.6 ± 9.2 years(Pooled Placebo);  2. Female: 29/51;  3. Race: Black or African American 22/51, Asian 16/51, White 13/51. | Agent: VIS649 (sibeprenlimab);  Dose: Single IV doses – 0.5, 2.0, 6.0, or 12.0 mg/kg;  Comparator: Placebo (0.9% saline);  Route: IV infusion over 1 hour;  Treatment duration: Single dose; follow-up 16–24 weeks;  Cohort 5: VIS649 6.0 mg/kg or placebo on Day 1, followed by tetanus/diphtheria vaccine on Day 28. | Safety and tolerability (AEs, labs, vitals, ECGs, physical exams). | 1. Pharmacokinetics (Cmax, AUC, t½, CL, Vd);   2. Pharmacodynamics (changes in serum APRIL, IgA, IgG, IgM, Gd-IgA1). |
| Sibeprenlimab  NCT04287985 | IgAN | Inclusion Criteria:  1. Adults (≥18 years) with biopsy-proven primary IgA nephropathy;  2. 24-hour urinary protein-to-creatinine ratio ≥ 0.75 g/g (or urinary protein ≥ 1.0 g/day);  3. eGFR ≥ 30 mL/min/1.73 m² (CKD-EPI 2009 equation);  4. Serum IgG ≥ 700 mg/dL, IgM ≥ 37 mg/dL, IgA ≥ 70 mg/dL;  5. Receiving highest stable dose of ACE inhibitor or ARB (minimal side effects) for ≥ 3 months before screening.  Exclusion Criteria:  1. Secondary IgA nephropathy or other coexisting causes of CKD;  2. Nephrotic syndrome;  3. Type 1 diabetes or uncontrolled type 2 diabetes;  4. Uncontrolled blood pressure;  5. Systemic immunosuppressive/glucocorticoid therapy within 16 weeks before screening;  6. Known chronic infectious disease;  7. Oxford MEST/MEST-C score of T2 or C2 (>50% tubulo-interstitial fibrosis or crescents in >25% of glomeruli). | 1. Median age (range): 39 years (18–73);  2. Male: 56.8%;  3. Race: Asian: 74.2%, White: 23.2%, Black: 0.6% ;  4.BMI: 27.6 ± 5.9 kg/m²;  5. History of hypertension: 72.3%;  6. Baseline UPCR ≤ 2.0 g/g: 63.9%; > 2.0 g/g: 23.9%;  7. Median baseline eGFR (range): 58.0 mL/min/1.73 m² (34–154) in pooled sibeprenlimab group; 68.5 mL/min/1.73 m² (33–116) in placebo group. | Agent: Sibeprenlimab ;  Doses: 2, 4, or 8 mg/kg IV;  Comparator: Placebo (saline);  Frequency: Once monthly for 12 months (total 12 doses);  Add-on to: Standardized ACE inhibitor/ARB therapy. | Change from baseline in log-transformed 24-hour urinary protein-to-creatinine ratio at Month 12. | 1. Change in UPCR at Months 9 and 16;  2. Clinical remission (urinary protein < 300 mg/day);  3. Change from baseline in eGFR at Month 12;  4. Pharmacodynamics (changes in serum IgA, IgG, IgM);  5. Safety (AEs, lab assessments, physical exams). |
| Sibeprenlimab  NCT05248646 | IgAN | Inclusion Criteria:  1. Adults (≥18 years) with biopsy-proven primary IgA nephropathy;  2. 24-hour urinary protein-to-creatinine ratio ≥ 0.75 g/g (or 24-hour urine protein ≥ 1.0 g/day);  3. eGFR ≥ 30 mL/min/1.73 m² (CKD-EPI 2021 creatinine equation);  4. Stable maximally tolerated ACE inhibitor or ARB for ≥3 months (or intolerant to both);  5. Stable SGLT2 inhibitor use ≥3 months if applicable.  Exclusion Criteria:  1. Secondary IgA nephropathy or other glomerular diseases;  2. Nephrotic syndrome;  3. Type 1 or uncontrolled type 2 diabetes;  4. Uncontrolled hypertension;  5. Systemic immunosuppressive therapy or glucocorticoids within 16 weeks before screening;  6. Known chronic infectious disease;  7. Oxford MEST/MEST-C score T2 or C2 (>50% fibrosis or crescents >25%);  8. Pregnancy or breastfeeding;  9. History of malignancy or severe allergic reactions. | 1. Median age (range), years: 42.0 (18–75)(Sibeprenlimab), 43.0 (18–83)(Placebo);  2. Male: 65.8%(Sibeprenlimab), 59.5%(Placebo);  3. Asian: 61.8%(Sibeprenlimab), 56.5%(Placebo);  4. Mean eGFR (mL/min/1.73 m²): 63.5(Sibeprenlimab), 63.4(Placebo);  5. Geometric mean UPCR (g/g): 1.33(Sibeprenlimab), 1.32(Placebo);  6. Median UPCR (range): 1.22 (0.49–6.69)(Sibeprenlimab), 1.28 (0.50–5.46)(Placebo);   7. Hematuria: 78.3%(Sibeprenlimab), 70.8%(Placebo). | Agent: Sibeprenlimab;  Dose: 400 mg subcutaneous;  Comparator: Placebo;  Frequency: Every 4 weeks;  Duration: 100 weeks (26 doses);  Add-on to: Supportive care (ACEi/ARB, SGLT2i as per background). | Change in 24-hour urinary protein-to-creatinine ratio at 9 months (week 40) compared with baseline. | 1. Annualized eGFR slope over 24 months; 2. Change in spot UPCR, hematuria, proteinuria remission;   3. Serum galactose-deficient IgA1 (Gd-IgA1), APRIL, IgA, IgG, IgM;  4. Safety (adverse events). |
| Zigakibart  NCT03945318 | IgAN | Inclusion Criteria (Part 3):   1. Adults (≥18 years) with biopsy-proven primary IgA nephropathy;   2. Total urine protein ≥ 0.5 g/24 h or UPCR ≥ 0.5 g/g;  3. eGFR ≥ 30 mL/min/1.73 m²;  4. On stable/optimized dose of renin-angiotensin system (RAS) inhibitor.  Exclusion Criteria (Part 3):  Not specified in detail in the provided text; refer to Supplementary Methods for full criteria | Part 3, N=40:  1. Median age (range), years: 38.5 (21–74);  2. Male: 70.0%;  3. Race: White / Asian: 60.0% / 32.5%;  4. Median eGFR (range), mL/min/1.73 m²: 66.5 (30–131);  5. eGFR < 60 mL/min/1.73 m²: 37.5%;  6. Median 24h urine protein (range): 1.1 (0.3–7.0)g/d;  7. Median 24h UPCR (range): 722.3 (210.5–4551.2)mg/g. | Agent: Zigakibart;  Dose in Part 3: 600 mg subcutaneous (SC) every 2 weeks (Q2W);  Cohort 1: 450 mg IV → 600 mg SC Q2W;  Cohort 2: 600 mg SC Q2W;  Treatment duration: Ongoing; 100-week data reported;  Route: Subcutaneous (SC) administration. | Safety and tolerability of zigakibart in healthy volunteers (Parts 1 & 2) and patients with IgAN (Part 3). | 1. Pharmacokinetics (PK) of zigakibart;  2. Immunogenicity (anti-drug antibodies);  3. Pharmacodynamics (PD): changes in immunoglobulins (IgA, IgG, IgM, Gd-IgA1);  4. Efficacy: change in proteinuria (UPCR), eGFR stabilization, hematuria resolution. |
| Telitacicept  NCT05799287 | IgAN | Inclusion Criteria:  1. Adults (≥18 years) with biopsy-proven primary IgA nephropathy;  2. Persistent proteinuria: 24-hour urinary protein-to-creatinine ratio ≥0.8 g/g (both in grams) OR 24-hour urine total protein ≥1.0 g/day;  3. eGFR ≥30 mL/min/1.73 m² (CKD-EPI equation);  4. On stable maximum tolerated/labeled ACE inhibitor or ARB for ≥4 weeks before randomization (background therapy ≥12 weeks);  5. SGLT2 inhibitors permitted if stable dose; systemic glucocorticoids/immunosuppressants prohibited (≥12-week washout).  Exclusion Criteria:  Not fully detailed in provided text; refer to Supplementary Appendix for complete criteria. | 1. Mean age (years): 37.8 ± 9.4(Telitacicept), 38.6 ± 10.0(Placebo);  2. Female: 51.6%(Telitacicept), 56.0%(Placebo);  3. Mean eGFR (mL/min/1.73 m²): 76.5 ± 26.9(Telitacicept), 74.4 ± 27.2(Placebo);  4. Median UPCR (range): 1.23 (0.47–5.86)g/g(Telitacicept), 1.29 (0.54–6.59)g/g(Placebo);  5. Hematuria: 71.1%(Telitacicept), 70.4%(Placebo);  6. Mean disease duration (months): 51.8 ± 52.5(Telitacicept), 47.2 ± 43.3(Placebo). | Agent: Telitacicept;  Dose: 240 mg subcutaneously;  Comparator: Matching placebo;  Frequency: Once weekly;  Duration: 39 weeks (Stage A; ongoing Stage B to 104 weeks);  Add-on to: Supportive care (ACEi/ARB ± SGLT2i). | Geometric mean ratio of 24-hour urinary protein-to-creatinine ratio at Week 39 relative to baseline. | 1. Geometric mean ratio of eGFR relative to baseline;  2. Geometric mean ratio of 24-hour urinary albumin-to-creatinine ratio;  3. ≥30% decline in eGFR;  4. UPCR <0.8 g/g. |
| Telitacicept  NCT04291781 | IgAN | Inclusion Criteria:  1. Adults (≥18 years) with biopsy-proven primary IgA nephropathy;  2. eGFR > 35 mL/min/1.73 m²;  3. Proteinuria ≥ 0.75 g/day despite optimal supportive therapy (ACEi/ARB for ≥3–6 months).  Exclusion Criteria:  Not fully detailed in provided text; refer to supplementary materials for complete criteria. | 1. Mean age (years): 37.0;  2. Female: 47.7%;  3. Mean BMI (kg/m²): 25.7;  4. Mean eGFR (mL/min/1.73 m²): 79.4;  5. Median urine protein (g/day): 1.66;  6. Median IgA (g/L): 3.02. | Agent: Telitacicept;  Doses: 160 mg or 240 mg subcutaneously;  Comparator: Placebo;  Frequency: Once weekly;  Duration: 24 weeks;  Add-on to: Supportive care (ACEi/ARB). | Mean change in 24-hour proteinuria from baseline at Week 24. | 1. Changes in eGFR, serum IgA, IgG, IgM levels;   2. Safety (adverse events). |
| Atacicept  NCT04716231 | IgAN | Inclusion Criteria:  1. Adults (≥18 years) with biopsy-confirmed IgA nephropathy;  2. 24-hour urinary protein-to-creatinine ratio ≥1.0 g/g (or proteinuria ≥1.0 g/day);  3. eGFR ≥30 mL/min/1.73 m²;  4. Stable renin-angiotensin system (RAS) inhibitor at maximum labeled/tolerated dose for ≥12 weeks;  5. Blood pressure ≤150/90 mm Hg;  6. Stable SGLT2 inhibitor dose for ≥12 weeks if applicable.  Exclusion Criteria:  1. Secondary IgA nephropathy;  2. Nephrotic syndrome within 6 months before screening;  3. Rapidly progressive glomerulonephritis;  4. Other specific criteria not detailed in provided text. | 1. Mean age (years): 40.1 ± 11.1 (Atacicept), 40.9 ± 11.6 (Placebo);  2. Male: 53.8% (Atacicept), 59.8% (Placebo);  3. Asian: 55.7% (Atacicept), 53.6% (Placebo);  4. Mean UPCR (g/g): 1.7 ± 0.9 (Atacicept), 1.8 ± 1.2 (Placebo);  5. Mean eGFR (mL/min/1.73 m²): 65.3 ± 27.7 (Atacicept), 64.9 ± 29.0 (Placebo);  6. Mean proteinuria (g/d): 2.2 ± 1.0 (Atacicept), 2.3 ± 1.4 (Placebo);  7. RAS inhibitor use: 99.1% (Atacicept), 100% (Placebo);  8. SGLT2 inhibitor use: 55.7% (Atacicept), 50.5% (Placebo). | Agent: Atacicept;  Dose: 150 mg subcutaneously;  Comparator: Matching placebo;  Frequency: Once weekly;  Route: Self-administered subcutaneously at home;  Duration: Ongoing; 36-week interim analysis reported; 104-week total planned. | Percentage change from baseline in 24-hour urinary protein-to-creatinine ratio. | 1. Change from baseline in serum galactose-deficient IgA1 (Gd-IgA1) level;  2. Resolution of hematuria;  3. eGFR change at Week 52 (not reported; blinded). |
| Atacicept  NCT02808429 | IgAN | Inclusion Criteria:  1. Age ≥ 18 years;  2. Biopsy-confirmed IgA nephropathy within 60 months before screening (Oxford-MEST classification);  3. Persistent proteinuria: UPCR ≥ 1.0 mg/mg by 24-hour urine collection (or ≥0.75 mg/mg if documented ≥1.0 mg/mg within 12 months);  4. On stable optimal ACEi and/or ARB therapy for ≥8 weeks before screening;  5. Up-to-date vaccinations against pneumococcus and influenza.  Exclusion Criteria:  1. Concomitant significant renal disease other than IgAN (e.g., diabetic nephropathy, lupus nephritis, Henoch-Schönlein purpura);  2. Serum IgG < 6 g/L;  3. 50% glomeruli with global glomerulosclerosis or >50% cortical tubular atrophy/interstitial fibrosis;  4. eGFR <25, <35, or <45 mL/min/1.73 m² depending on biopsy timing;  5. Blood pressure >140/90 mmHg;  6. Active infection or high infectious risk;  7. Prior cyclophosphamide, concomitant immunosuppressants, or prior B-cell-directed biologics (e.g., rituximab). | 1. Mean age (years): 46 ± 3.1(Placebo), 41 ± 16.9 (Atacicept 25 mg), 43 ± 8.9 (Atacicept 75 mg);  2. Female: 50%;  3. Race: White: 11 (68.8%), Asian: 3 (18.8%), Other: 2 (12.5%), Black: 0;  4. Median eGFR (mL/min/1.73 m²): 49 (Placebo), 57 (Atacicept 25 mg), 55 (Atacicept 75 mg);  5. Median UPCR (mg/mg): 1.6, 1.8, 1.4;  6. Mean Gd-IgA1 (ng/mL): 7690 ± 3642, 6258 ± 3211, 6052 ± 2773. | Agent: Atacicept;  Doses: 25 mg or 75 mg subcutaneously;  Comparator: Placebo;  Frequency: Once weekly;  Duration: Planned 72 weeks; early termination due to slow enrollment; patients completed ≥48 weeks. | Safety and tolerability (TEAEs, serious AEs, AEs leading to discontinuation, AESIs). | 1. Pharmacodynamics: changes in serum IgA, IgG, IgM, Gd-IgA1, complement C3/C4;  2. Clinical efficacy: change in proteinuria (24-hour UPCR) and eGFR over time. |
| Povetacicept  NCT05732402 | IgAN | Inclusion Criteria:  1. Adults (≥18 years) with autoantibody-associated glomerular disease;  2. Biopsy-confirmed IgAN within 10 years;  3. UPCR ≥0.5 g/g;  4. eGFR ≥30 mL/min/1.73 m² (CKD-EPI 2021).  Exclusion Criteria:  1. Background immunosuppressive therapy prohibited;  2. Full criteria listed in Supplementary Material. | 1. Mean age (years): 47.6 ± 11.7(Povetacicept 80 mg) 45.1 ± 12.1(Povetacicept 240 mg);  2. Male: 33%, 55%;  3. Asian: 48%, 55%;  4. Mean UPCR (g/g): 1.3 ± 0.7, 1.2 ± 0.8;  5. Mean eGFR (mL/min/1.73 m²): 76.9 ± 34.0, 63.5 ± 29.5;  6. ACEi/ARB use: 86%, 100%;  7. SGLT2i use: 29%, 45%;  8. Gd-IgA1 (ng/mL): 9068 ± 4324, 7251 ± 3606. | Agent: Povetacicept;  Doses: 80 mg or 240 mg subcutaneously;  Frequency: Every 4 weeks (Q4W);  Duration: Up to 104 weeks (initial 24 weeks + extensions). | Safety (TEAEs, labs, vital signs, ECGs). | 1. Change in 24-hour UPCR;  2. Change in eGFR;  3. Clinical remission: UPCR <0.5 g/g + negative hematuria + eGFR decline <25%;  4. Hematuria resolution (moderate/large → negative/small);  5. Pharmacodynamics: Gd-IgA1 reduction. |
| Belimumab  NCT01639339 | LN | Inclusion Criteria:   1. Age ≥ 18 years; 2. Autoantibody-positive SLE (ANA ≥ 1:80 and/or anti-dsDNA antibodies); 3. Fulfilled 1982 ACR classification criteria for SLE (updated 1997); 4. Urinary protein-to-creatinine ratio ≥ 1.0 at screening; 5. Biopsy-proven lupus nephritis (ISN/RPS class III, IV, with or without V, or pure class V) within 6 months before or during screening; 6. Active or active/chronic lesions on biopsy;   7. Induction therapy initiated within 60 days before randomization.  Exclusion Criteria:   1. Dialysis within 1 year; 2. eGFR < 30 mL/min/1.73 m²; 3. Prior failure of both cyclophosphamide and mycophenolate mofetil induction; 4. Cyclophosphamide induction within 3 months before trial;   5. B-cell–targeted therapy (including belimumab) within 1 year before randomization. | 1. age: 33.4 ± 10.6 years;  2. Female: 88%;  3. Race: Asian 50%, White 33%, Black 14%;  4. Median lupus nephritis duration: 0.2 years (IQR 0.1–3.3);  5. Biopsy class: III/IV 58%, III/IV+V 26%, pure V 16%;  6. Mean UPCR: 3.4 ± 3.2;  7. Mean eGFR: 100.5 ± 40.2 mL/min/1.73 m²;  8. Mean SLEDAI-2K: 12.3 ± 5.0;  9. Anti-dsDNA positive: 77%. | Agent: Belimumab (recombinant human IgG-1λ monoclonal antibody);  Dose: 10 mg/kg intravenous;  Comparator: Matching placebo;  Background standard therapy: MMF or CYC-AZA;  Treatment duration: 104 weeks (double-blind period). | Primary renal efficacy response (PERR) at 104 weeks: 1. UPCR ≤ 0.7;  2. eGFR ≥ 60 mL/min/1.73 m² or no worse than 20% below pre-flare value;  3. No rescue therapy. | 1. PERR at week 52;  2. Time to renal-related event or death;  3. Ordinal renal response without urinary sediment at week 104;  4. Complete Renal Response at week 104: UPCR < 0.5 g/g; eGFR ≥ 90 mL/min/1.73 m² or no worse than 10% below pre-flare value; No rescue therapy. |
| Belimumab  NCT02260934 | LN | Inclusion Criteria:   1. Age ≥ 18 years; 2. Met ACR or SLICC criteria for SLE; 3. Positive ANA and/or anti-dsDNA at screening; 4. Recurrent or refractory LN; 5. Prior treatment with cyclophosphamide (CYC) or mycophenolate mofetil (MMF); 6. UPCR > 1.0 (24-hour urine);   7. Kidney biopsy within 18 months showing ISN/RPS Class III, IV, or III/IV + V.  Exclusion Criteria:   1. Prior rituximab at any time; 2. Other B-cell biologic therapy within 12 months;   3. Active infection or other significant medical conditions per investigator discretion. | 1. Age: RC 32.3 ± 11.4 yrs, RCB 34.5 ± 9.1 yrs;  2. Female: RC 81.8%, RCB 90.5%;  3. Race: White 16/43, Black 18/43, Asian 5/43;  4. Mean UPCR (24h): RC 3.4 ± 1.5; RCB 3.3 ± 2.5;  5. Mean eGFR: RC 92.7 ± 36.0; RCB 89.1 ± 33.9 mL/min/1.73m²;  6. Anti-dsDNA positive: RC 90.9%; RCB 90.5%;  7. Hypocomplementemia (C3): RC 81.8%; RCB 76.2%. | All participants (induction):  1. Methylprednisolone 100 mg IV (Week 0, 2);  2. Rituximab 1000 mg IV (Week 0, 2);  3. Cyclophosphamide 750 mg IV (Week 0, 2);  4. Prednisone 40 mg/day taper to ≤10 mg/day by Week 12, continued through Week 96.  Randomization at Week 4:  1. RC group: No additional treatment;  2. RCB group: Belimumab 10 mg/kg IV at Weeks 4, 6, 8, then every 4 weeks through Week 48.  Total follow-up: 96 weeks. | Proportion of participants with ≥1 Grade 3 or higher infectious adverse event by Week 48 (Safety). | 1. B-cell reconstitution (return to baseline or LLN);  2. Grade 4 hypogammaglobulinemia (IgG < 300 mg/dL) with Grade ≥3 infection;  3. Efficacy (prospectively defined):  3.1 Complete Renal Response (CR): UPCR < 0.5; eGFR ≥120 or >80% of baseline; prednisone adherence;  3.2 Partial Renal Response (PR): UPCR improvement ≥50% from baseline; same eGFR and steroid criteria. |
| Atacicept  NCT00573157 | LN | Inclusion Criteria:  1. SLE diagnosis (≥4 ACR criteria);  2. Positive ANA (≥1:80) and/or anti-dsDNA (≥30 IU/mL);  3. Renal biopsy within 12 months showing active LN (ISN/RPS Class III or IV);  4. Active LN: proteinuria (UPr:Cr >1.0 mg/mg) and hematuria (>10 RBCs/hpf with/without casts);  5. Calculated GFR >30 mL/min/1.73 m² at screening.  Exclusion Criteria:  1. Renal disease unrelated to SLE;  2. Calculated GFR ≤30 mL/min/1.73 m² at screening;  3. Comorbidities requiring corticosteroids;  4. Progressive disease (≥25% decrease in GFR or doubling of UPr:Cr between screening and randomization). | 1. Age range: 18–54 years;  2. Female: 66.7% (4/6);  3. Race: African American (5), Caucasian (1), Asian (1);  4. All patients had Class IV LN;  5. Baseline proteinuria: More severe in atacicept group (UPr:Cr ≥3.0 vs ≤2.3 mg/mg);  6. Mean GFR at screening: Lower in atacicept group (52.1 vs 76.1 mL/min/1.73 m²). | Agent: Atacicept;  Dose: 150 mg subcutaneously;  Regimen: Twice weekly for 4 weeks, then once weekly;  Comparator: Placebo.  Background therapy (all patients):  1. MMF: initiated at 1 g/day (Day –14), increased to 3 g/day by Day 1;  2. Corticosteroids: prednisone 0.8 mg/kg/day (max 60 mg/day) starting Day –14, tapered from Week 5. | Efficacy and safety of atacicept + MMF + corticosteroids in active LN. | Key Discontinuation Criteria:  1. IgG <3 g/L;  2. ≥25% decrease in GFR or doubling of UPr:Cr;  3. No improvement at Week 24. |
| Belimumab  NCT01610492 | pMN | Inclusion Criteria  1. Age 18–75 years;  2. Biopsy-proven PMN;  3. Nephrotic-range proteinuria for >3 months despite supportive therapy (ACEi/ARBs);  4. Positive anti-PLA2R autoantibodies (PLA2R-Ab).  Exclusion Criteria.  1. Secondary causes of membranous nephropathy;  2. Uncontrolled hypertension;  3. eGFR < 40 mL/min/1.73 m²;  4. Corticosteroids >10 mg/day or other immunosuppressants within appropriate washout period. | 1. Median age: 48 years (range 24–69);  2. Male: 11/14 (79%);  3. Median disease duration: 6 months (range 4–29);  4. Median uPCR: 831 mg/mmol (range 321–1221);  5. Median 24h protein excretion: 11.7 g (range 4.0–20.4);  6. Median eGFR: 78 mL/min/1.73 m² (range 43–112);  7. Median PLA2R-Ab: 168 RU/mL (range 17–994);  8. Median serum albumin: 23 g/L (range 16–37);  9. Prior immunosuppressive therapy: 3/14 (21%). | Agent: Belimumab;  Dose: 10 mg/kg intravenous infusion;  Frequency: Every 4 weeks up to Week 100 (or 3 months after complete remission if earlier);  Modified dosing: Participants with uPCR >1000 mg/mmol were dosed every 2 weeks;  Treatment duration: Up to 2 years; follow-up 6 months after last dose. | The co-primary endpoints were change from baseline in proteinuria (uPCR) and PLA2R-Ab at Week 28. | 1. Changes in proteinuria and PLA2R-Ab at other time points;  2. Incidence of partial remission (PR) and complete remission (CR);  3. Changes in eGFR, serum albumin, cholesterol, IgG, B-cell subsets, and pharmacokinetics. |
| Povetacicept  NCT05732402 | pMN | Inclusion Criteria:  1. Adults (≥18 years) with autoantibody-associated glomerular disease;  2. Historical biopsy-confirmed pMN;  3. UPCR ≥1.0 g/g;  4. Positive aPLA2R (≥14 RU/mL) or positive THSD7A autoantibody;  5. Inadequate proteinuria reduction despite ≥12 weeks of optimal supportive care;  6. eGFR ≥30 mL/min/1.73 m².  Exclusion Criteria:  Background immunosuppression prohibited except stable calcineurin inhibitor (optional). | 1. Mean age (years): 58.2 ± 18.1;  2. Male: 80%;  3. Race: Black or African American: 40% White: 40% Asian: 20%;  4. UPCR (g/g): 3.8 ± 1.9;  5. eGFR (mL/min/1.73 m²): 78.1 ± 24.9;  6. aPLA2R (RU/mL): 139.5 ± 92.1. | Agent: Povetacicept;  Dose: 80 mg subcutaneously Q4W;  Duration: Up to 104 weeks;  Background: Stable calcineurin inhibitor permitted. | Safety. | 1. Change in 24-hour UPCR;  2. Change in eGFR;  3. Complete remission: UPCR <0.5 g/g;  4. Partial remission: UPCR reduction >50% and <3.5 g/g;  5. Immunologic remission: aPLA2R <14 RU/mL;  6. Pharmacodynamics: aPLA2R reduction. |
